# A Standardised Combinational Method for Evaluating Antimicrobial Compounds Against Biofilm Attachment, Development and Eradication

**DOI:** 10.3390/microorganisms14061238

**Published:** 2026-05-30

**Authors:** Kevin Masterson, Mark Lynch, Ian Major, Neil Rowan

**Affiliations:** 1 Faculty of Science and Health, Technological University of the Shannon: Midlands Midwest, N37HD68 Athlone, Ireland; 2 Polymer, Recycling, Industrial, Sustainability and Manufacturing Research Institute (PRISM), Technological University of the Shannon: Midlands Midwest, N37HD68 Athlone, Ireland; 3 Centre for Sustainable Disinfection and Sterilization, Technological University of the Shannon: Midlands Midwest, N37HD68 Athlone, Ireland; 4 CÚRAM Research Ireland Centre for Medical Device Research, University of Galway, H91W2TY Galway, Ireland

**Keywords:** biofilm disruption, bioactives, AMR bacteria, disease prevention

## Abstract

Biofilm-mediated antimicrobial resistance remains a significant challenge for healthcare and patient safety. Currently, there are gaps in standardised methods for assessing antimicrobials against biofilm formations such as (1) assessment of initial bacterial attachment inhibition, as well as (2) assessment of antimicrobial compounds against both the external biofilm mass and biofilm-embedded metabolically active bacteria. The aim of this study is to address these gaps by combining several anti-biofilm techniques. In the procedure96-well anti-biofilm assessments were performed using plate well and lid peg growth surfaces so as to determine the effects of bioactive compounds (silver nitrate (AgNO_3_), nisin, chitosan and zinc oxide nanopowder (ZnO)) on biofilm growth inhibition, formed biofilm reduction and bacterial attachment inhibition. These studies focused on the initial attachment stage against in vitro biofilms of *P. aeruginosa* and *S. aureus*. Effects were measured against biofilm mass using Crystal Violet (CV) staining, while embedded bacteria metabolic activity was measured using Resazurin. AgNO_3_ exhibited significant inhibition and reduction against *P. aeruginosa* at all stages of biofilm development (*p* < 0.0001). AgNO_3_ showed significant results against *S. aureus* during biofilm development and against the embedded, metabolically active population of established biofilms (*p* < 0.0001). Nisin showed significant inhibition against *S. aureus* biofilm populations (*p* < 0.0001). Chitosan showed significant increases in *S. aureus* biofilm formations following exposure, during initial attachment (*p* < 0.02), during biofilm growth (*p* < 0.0001) and against formed biofilm populations (*p* < 0.0001). ZnO showed significant increases during initial attachment exposure (*p* < 0.0001), but also exhibited growth inhibition (*p* < 0.0001) and biofilm reduction (*p* < 0.0001). Although variance in anti-biofilm efficacy was evident depending upon treatment used, Gram-staining phenotype and test growth surfaces, this combinational method offers potential for high throughput screening and for evaluating pipeline bioactives isolated from different environments for biofilm prevention, inhibition and removal. Additionally, this approach will help elucidate the relationship between bacteria of interest and biofilm mitigation.

## 1. Introduction

As the threat of antibiotic and antifungal drug resistance continues to globally rise, there is a pressing need for the development of alternative treatments to counteract this challenge [1,2,3]. While certain bacterial species have been identified to hold intrinsic antibiotic resistance through genetic variation, another major mechanism of resistance is mediated through biofilm formation that functions as a physical barrier against all anti-bacterial treatments, including antibiotics [4].

Biofilms are the product of close-knit bacterial communities which cooperate together to increase their survival. Biofilm formation can be described through four major phases, (1) attachment, (2) growth, (3) maturation and (4) detachment, after which the cycle repeats [5]. The attachment phase involves a primary bacterial species adhering to a surface and establishing a micro-colony. Following colony formation, attachment of the bacteria to the surface begins and, thereafter, formation of the biofilm matrix. This biofilm process is initiated through Quorum Sensing (QS) and exposure to external stress factors. The bacterial colony excretes an extracellular matrix in the form of extracellular polymeric substances (EPS) comprising a variety of compounds such as polysaccharides, lipids, proteins and nucleic particles [6]. The EPS matrix gives basic structure to the biofilm, but it also provides a physical barrier. In many instances, biofilms are regarded as multispecies, with the initial EPS formation being produced by several bacterial micro-colonies. The latter ultimately combine into the mature biofilm, with the initial secretion primarily activated in response to a high bacterial population density, as determined through the aforementioned QS [7,8].

During the bacterial growth phase, the biofilm expands, and embedded cells multiply. As the biofilm develops, it matures and begins to gain structure. The structure, as well as composition of the biofilm, varies depending on the bacterial species. For example, *Pseudomonas aeruginosa* forms mushroom-like micro-colonies, whereas *Streptococcus pneumoniae* forms tower-like assemblies [9]. After irreversible attachment, the biofilm initiates growth. The final stage involves detachment of small segments of the biofilms and release of planktonic bacterial cells from the matrix. These planktonic cells may then relocate and colonise other surfaces, giving further indication of the advanced pathology of biofilm-associated infections [10].

Biofilm formations are found in various medical fields, the majority of which occur on inorganic surfaces (such as medical devices, implants, catheters), but they may also develop on organic surfaces (such as plaque formations on teeth, endocarditis in the heart and mastitis in mammary glands) [11]. It is estimated that a majority of bacterial infections have been associated with biofilm formations [12]. As such, biofilm infections are frequently encountered and yield numerous challenges that greatly impede successful treatment. Biofilms confer a higher level of resilience to the containing bacterial species against external threats, such as the hosts’ immune response, antimicrobial compounds from other unrelated bacteria or medical treatments (such as antibiotics).

There are a number of different means through which biofilms can increase anti-microbial resistance of microorganisms (AMR). Although biofilm composition and their structures can vary between bacterial species, the cellular and molecular mechanisms aiding AMR are consistent, including EPS matrix structure, genotypic alterations preventing interaction with target ligands, high-density cell populations, QS and multi-drug efflux pumps [13,14]. The EPS matrix can physically hinder antibiotic diffusion, but it can also alter or degrade antibiotics via enzymes produced by embedded bacteria such as β-lactamase [15]. Additionally, the multi-drug efflux pumps of biofilm-embedded bacterial cells can effectively remove antimicrobial compounds from the internal environment of a biofilm [4].

Anti-biofilm assays have been used for several years to determine antimicrobial efficacy against various biofilm-forming bacteria [16]. Such methods typically assess potential antimicrobials by determining the lowest concentration required to inhibit biofilm formation (the minimum biofilm inhibitory concentration (MBIC)) or to eliminate an already developed biofilm (the minimum biofilm eradication concentration (MBEC)) [17,18]. In this present study, we describe an additional step for determining the concentration required to inhibit the initial primary bacterial attachment to a surface. Primary bacterial attachment is considered the most crucial point of biofilm formation, and assessment of a compound’s ability to prevent this occurrence would allow insight into its capacity to prevent and negate biofilm development. Previous studies have assessed treatment exposure during this bacterial attachment stage; however, they do not allow biofilm development to continue, with a focus on quantifying attached bacterial cells [19]. The understanding of how treatment exposure affects attachment and subsequent biofilm development would provide important data for surface treatments and early-stage intervention in order to prevent biofilm formations.

The development and application of the most appropriate methods to determine the effects of antimicrobials is important. For example, while the use of sonication has been extensively reported in order to detach bacterial cells from a surface, this physical approach varies in its efficacy to remove all cells from the biofilm [20,21]. Additionally, sonication has been found to cause cellular death during bacteria harvesting from biofilm formations [21,22]. Such issues can lead to misinterpretation of anti-biofilm treatment efficacy due to lower numbers of viable cells being determined, thus potentially over-estimating a treatment’s efficacy. The use of the redox indicator resazurin (Rez) bypasses these shortcomings by allowing measurement of bacteria population health without causing stress or disruption to the cells or biofilms [23]. While the low molecular weight and high water solubility of resazurin facilitate its diffusion through the EPS matrix, this standard assay primarily reflects the activity of cells near the biofilm periphery. Deeply embedded, non-metabolically active sessile bacteria and persister cells exhibit minimal respiration and may evade detection by redox-based dyes [24]. As Rez staining does not disrupt biofilm formations and is non-toxic, this allows the assay to be carried out prior to crystal violet (CV) staining of the biofilm mass, thus enabling he combined use of both methods for greater data generation from a single sample set.

As we previously demonstrated, four chosen bioactive molecules, silver nitrate (AgNO_3_), nisin, chitosan and zinc oxide nanopowder (ZnO), were shown to effectively inhibit bacterial growth of antimicrobial-resistant (AMR) strains of MRSA, VRE, *L. monocytogenes*, *E. coli* and *A. baumannii* [25]. Leveraging the findings from these bioactive studies, this present study aims to address biofilm formation with the intention of tackling biofilm-mediated resistance (BMR) and its ability to prevent microbial attachment, biofilm growth, and to eradicate already formed biofilms. The anti-biofilm capabilities of AgNO_3_, nisin, chitosan and ZnO will be assessed against *S. aureus* and *P. aeruginosa*. These are known biofilm-forming microorganisms and are used in this study as indicative representatives for Gram-positive and Gram-negative bacterial species. The use of *P. aeruginosa* and *S. aureus* as model bacteria is already an established and robust approach for initial antimicrobial screening for anti-biofilm assays, as these species are the most commonly found pathogens isolated from chronic wounds and respiratory infections [26,27]. While the use of both species in a single-biofilm model (i.e., a mixed-species model) would yield important results with regard to clinical research models, there are a number of identified drawbacks for this. It has been reported that *P. aeruginosa* frequently outcompetes *S. aureus* under the same cultures and biofilm models [28,29]. Mixed-biofilm models are reported to have high variability between replicates, while single-species models allow stable, proof-of-concept testing before progressing to more complex models. This was an important consideration due to the introduction of the attachment inhibition assessment step, the use of both plate wells and lid pegs, and also for initial assessment of the anti-biofilm capabilities of AgNO_3_, nisin, chitosan and ZnO. While there are numerous publications of biofilm assays, the primary focus of this study is to determine biofilm inhibition and eradication capabilities for each of these bioactive combinations [23,30,31,32,33,34]. Established standard protocols do not fully address all conditions for biofilm testing in clinical research, such as the use of different growth surfaces or effectively mimicking the behaviour of in vivo infections (i.e., pre-treatment with plasma) [17,31]. Additionally, some biofilm studies lack specificity of treatment exposure to the bacterial attachment stage, such as exposure for 24 h that would prevent differentiating attachment inhibition to biofilm inhibition. Other studies do not assess the effects that treatment exposure to attaching bacteria may have upon biofilm development (i.e., do not allow biofilm growth following treatment) [19,35].

With these considerations, the procedures used in this study have been developed to incorporate what can be considered important test conditions and parameters of assessment so as to determine the most successful conditions for both producing biofilms and also for assessing the effect of treatments [16,23,36,37]. Furthermore, while definite endpoints such as the MBIC and MBEC are standard in clinical biofilm research, the methods utilised in the present study cannot conform to these strict end points. Rather than defining the lowest concentration that can inhibit biofilm growth or eradicate a mature biofilm, this current study reports on the effects of each bioactive against bacterial attachment, biofilm inhibition and biofilm eradication in a dose-response fashion. The results of these assays will be determined by use of Rez indicator that supports analysis of the bioactives effects against biofilm-embedded, metabolically active bacterial populations. Additionally, the use of the CV stain provided insight into biofilm formation and mass.

## 2. Materials and Methods

### 2.1. Bacterial Culture Preparation

*Pseudomonas aeruginosa* (ATCC 27853) *and Staphylococcus aureus* (ATCC 29213) from American Type Culture Collection (LGC Standards, Middlesex, UK) were used. Strains were chosen, as they are well known biofilm-forming bacteria, which become much more tolerant to antibiotics such as ceftazidime, cefepime, imipenem, meropenem, ciprofloxacin, amikacin, erythromycin, clindamycin and rifampin once established within a biofilm [38,39,40,41]. Inoculum preparation methods were adapted from previously published protocols [20,42]. Lyophilized cultures were recovered as per ATCC protocol using tryptone soy broth (TSB) and streaking onto tryptone soy agar (TSA) and incubated at 37 °C overnight. Individual colonies were used to inoculate Microbank™ vials (Pro-Lab Diagnostics, Merseyside, UK), as per their documentation, and stored at −80 °C. Prior to use, the bacterial strain was revived on TSA overnight at 37 °C to prepare a subculture. The resulting subculture was then used to inoculate secondary subcultures for use in inoculum preparation. Several bacterial colonies of similar morphology were taken and suspended in Brain Heart Infusion (BHI) broth. The bacterial suspension was incubated in a rotary incubator at 37 °C, 120 RPM. Bacterial growth was monitored by taking 1 mL aliquots from the suspension and measuring the absorbance at 625 nm (Abs_625_). Absorbance was measured hourly until a reading ≥0.25 Abs_625_ was noted, which represents approximately 3 × 10^8^ cfu/mL. This liquid culture was then diluted using BHI broth to final desired inoculum concentration depending on assay being carried out, for final in-well bacterial cell density of 1 × 10^6^ cells/mL.

### 2.2. Bioactive Solution Preparation

Silver nitrate (AgNO_3_) (SKU: S8157, CAS: 7761-88-8), nisin, 2.5% (SKU: N5764, CAS: 1414-45-5), Chitosan, low molecular weight (SKU: 448869, CAS: 9012-76-4), zinc oxide (ZnO) (nanopowder: <100 nm particle size (SKU: 544906, CAS: 1314-13-2)) were purchased from Sigma-Aldrich/Merck (Darmstadt, Germany). The bioactive solutions were prepared as per previously published materials [25]. In brief, AgNO_3_ was prepared in a solution of 28% (*v*/*v*) PEG-400 and 26% (*w*/*v*) d-sorbitol [43]. The sample was vortexed to ensure complete solubility, filter-sterilised using a 0.2 µm syringe filter and stored at 4 °C. Nisin was dissolved in a solution of 400 mM NaCl, adjusted to pH 3.25 with 0.1 M HCl. Chitosan was dissolved using 1% (*v*/*v*) acetic acid with stirring overnight at 50 °C to ensure complete solubilisation and then adjusted to pH 5.5 with 0.4 M NaOH. ZnO was suspended in dH_2_O, vortexed vigorously. These preparations were sterilised by autoclave before use.

### 2.3. Biofilm Assay Set up

All steps were carried out aseptically using sterile materials, unless otherwise stated. Anti-biofilm potential of each bioactive was carried out using two chosen bacterial strains, *P. aeruginosa* and *S. aureus*, per methods adapted from previously published protocols which varied in their execution, with different studies utilising plate wells or lid pegs, and CV or Rez analysis [23,31,44,45]. Biofilm assays were carried out in Thermo Scientific™ Nunc™ (Waltham, MA, USA) 96-well plates using Thermo Scientific™ Nunc™ Immuno TSP peg lids. Plate wells and lid pegs were coated with a bovine plasma solution before use to aid attachment. Bovine plasma (citrated and lyophilized) was purchased from Merck (Product code: P4639). Thermo Scientific™ Nunc™ flat-bottom plates (non-treated) (Product code: 10000571) and Thermo Scientific™ Nunc™ Immuno TSP lids (Product code: 10429962) were purchased from Thermo Fisher Scientific. The bovine plasma solution was diluted to a 1% solution in PBS with 120 μL aliquoted to each well and a TSP lid placed on the plate, submerging the pegs in the 1% bovine plasma solution in the wells. The plate was then incubated at 4 °C overnight. Before use, the 1% plasma solution was aspirated, and the wells and pegs were briefly washed in PBS.

Setups for assessments of attachment inhibition, biofilm inhibition and biofilm reduction followed similar steps; however, they differed by the point at which treatments were applied. Columns 1–10 were treated using serially diluted treatment solution, with two rows per treatment (duplicates), leaving column 11 to act as the negative control (i.e., 0% inhibition/reduction) and as Growth Control (GC) (i.e., 100% growth). Column 12 was left without treatment and inoculum, acting as the positive control (i.e., 100% inhibition) and as Sterility Control (SC).

Bacterial inoculum adjusted to 1 × 10^6^ cells/mL was added to wells, pegs inserted, and plate incubated at 37 °C for 1 h to allow initial attachment. During attachment inhibition assays, treatment solutions were added at this stage prior to bacterial inoculum, which was adjusted to account for this and give a final in well concentration of 1 × 10^6^ cells/mL. Plates contents were aspirated, the wells and pegs were washed in PBS to remove loosely attached bacterial cells, and treatments were conducted if applicable. Fresh BHI broth was added, pegs re-inserted and plates incubated under static conditions for 24 h at 37 °C. During biofilm inhibition assays, treatment solutions were added during this stage. Following incubation, plate contents were aspirated, wells and pegs washed in PBS. For attachment inhibition and biofilm inhibition assays, plates and pegs are prepared for Rez and CV analysis as per Section 2.4 and Section 2.5 below. For biofilm reduction assays, fresh BHI was added to wells with serially diluted treatment solutions, pegs re-inserted were and plate was incubated under static conditions for a further 24 h at 37 °C, after which contents were aspirated, wells and pegs washed with PBS and then were prepared for Rez and CV analysis.

### 2.4. Resazurin Analysis

Following final incubations, a Rez solution (10 µg/mL) prepared in BHI was added to each well of the biofilm plate and to each well of a separate sterile 96-well plate. The biofilm plate was closed with a standard 96-well plate lid, and the pegs were placed into the wells of the new plate. The fluorescence of both plates was measured using a Synergy HT microplate reader (Excitation: 528/20, Emission: 590/35) for time-point 0 (t = 0), and both plates were covered and incubated at 37 °C, 120 RPM. Fluorescence was measured each hour until a noticeable fluorescence signal was measured in every well or up to a max incubatory period of 5 h. Analysis of results determined the optimal incubatory time for *P. aeruginosa* to be 4 h (t = 4) and *S. aureus* to be 2 h (t = 2). Rez conversion readings were used to determine overall metabolic activity within the biofilms of each plate well or lid peg. This was calculated by subtracting the blank Rez readings (t = 0) from each well reading and then normalizing treatment wells results against the averaged growth control readings (0% reduction). Lower conversions represent a lower population of metabolically active bacteria within the biofilm.

### 2.5. Crystal Violet Analysis

During CV analysis preparation, steps were no longer required to be carried out aseptically. Following Rez analysis, plate contents were emptied, and the wells and pegs were washed with dH_2_O. The biofilms were heat-fixed in place to prevent sample loss by putting the biofilm plate and peg lid to a 60 °C oven overnight. Following heat-fixing, 120 μL 0.1% CV solution was added to each well of the biofilm plate, and the lid pegs were placed into the wells. The plate was left to incubate at room temperature (RT) for 10–15 min. The CV solution was removed, and the wells and lid pegs were washed in dH_2_O until no more CV was being evidently washed off. At this point, formed biofilms were evident by purple staining on the pegs or around the well edges (see Figure S1 for examples of stained biofilms on both peg lids and plate wells). Both plate and lid were then left to air dry in a fume hood. The CV was solubilised using 30% acetic acid (AcOH) by adding 120 μL to each well of the biofilm plate and to each well of a separate 96-well plate for the peg lid. Both plates were incubated under rotation for at least 30 min to allow complete solubilisation. If plates and pegs were not observed to have complete solubilisation of the CV, they were incubated for a further 30 min. Absorbance of the solubilised CV in each well was read at 590 nm using a Synergy HT microplate reader (Winooski, VT, USA). The CV absorbance was used to quantify the outer biofilm formation on the lid pegs and plate wells, calculated by subtracting the averaged blank absorbance readings (SC, 0% growth) from averaged treatments and normalising against the GC wells (100% growth). Results were used to determine final attachment inhibition, biofilm inhibition and biofilm reduction by assessing the reduced metabolically active cell numbers or reduced biofilm formations.

### 2.6. Statistical Analysis and Data Visualisation

Experiments were carried out across four biological replicates. The normalized % results from Rez and CV analyses were input to GraphPad Prism 8.0.1 for Windows (GraphPad Software, San Diego, CA, USA) for statistical analysis and data visualisation. Data were visualised by use of an XY Scatter Plot and analysed using a Nonlinear regression with [inhibitor] vs. normalized response–Variable slope model, with outliers detected and eliminated using ROUT method (Q = 1%), constrained to Hillslope = 1.0. The cleaned data were plotted on an XY scatter plot, with Concentration (µg/mL) (*X*-axis) vs. % Inhibition (*Y*-axis). The cleaned data were further analysed for significance by use an ordinary One-Way ANOVA with Dunnett’s test, comparing individual normalized % results for each treatment concentration against the corresponding no treatment control response (GC), expressed in terms of a *p* value following Graph Pad (GP) style (**** *p* < 0.0001, *** *p* < 0.001, ** *p* < 0.01, * *p* < 0.05).

## 3. Results

### 3.1. Attachment Inhibition

#### 3.1.1. AgNO_3_ Attachment Inhibition

**Figure 1:**
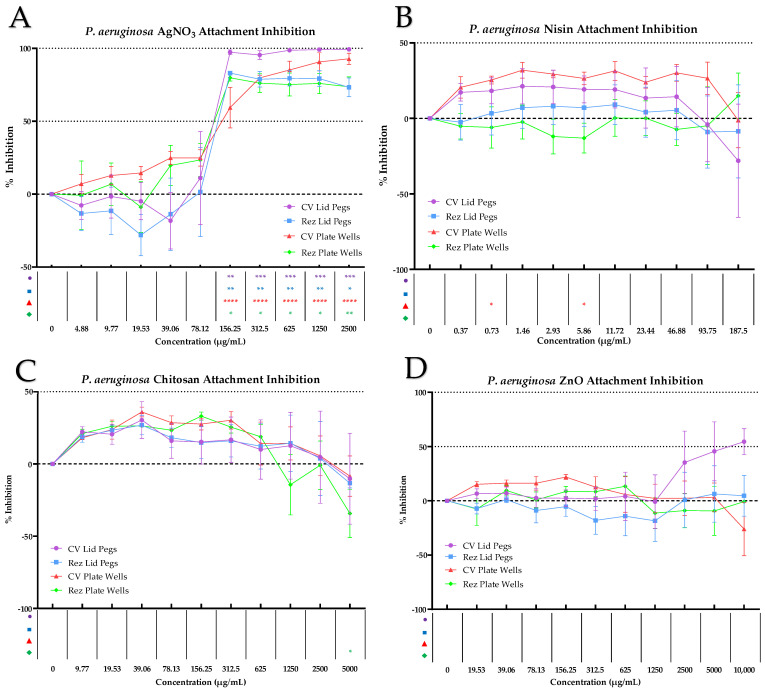
Evaluation of *P. aeruginosa* Attachment Inhibition. Dose-response XY scatter plots presenting four bioactives of (**A**) = AgNO3, (**B**) = Nisin, (**C**) = Chitosan, (**D**) = ZnO at serially diluted concentrations (*X*-axis) against *P aeruginosa %* attachment inhibition (*Y*-axis). Each graph shows effects, as determined by crystal violet (CV) and resazurin (Rez), from biofilm growth on plate wells and lid pegs. % Inhibition was calculated by normalising the raw data against the average response of the positive (100% inhibition) and negative (0% inhibition) controls. Data points were fitted with a nonlinear regression model; outliers were identified and removed using the ROUT method (Q = 1%). Significance was determined by use of an ordinary One-Way ANOVA with Dunnett test and is shown by asterisks in GP style (**** *p* < 0.0001, *** *p* < 0.001, ** *p* < 0.01, * *p* < 0.05). Error bars represent the standard error of the mean (SEM) from four independent biological replicates (*n* = 4).

AgNO_3_ exhibited significant attachment inhibition against *P. aeruginosa*, as determined by CV analysis at concentrations of 2500 µg/mL (99.29% *p* = 0.0004), 1250 µg/mL (99.13% *p* = 0.0004), 625 µg/mL (98.71% *p* = 0.0004), 312.5 µg/mL (95.46% *p* = 0.0006), and 156.25 µg/mL (97.34% *p* = 0.0012) on the lid pegs (Figure 1A). Rez analysis also identified significant attachment inhibition at concentrations of 2500 µg/mL (73.31% *p* = 0.015), 1250 µg/mL (79.27% *p* = 0.0074), 625 µg/mL (79.45% *p* = 0.0072), 312.5 µg/mL (78.86% *p* = 0.0078), and 156.25 µg/mL (83.03% *p* = 0.0098).

Very similar results were observed on the plate well biofilm growth, with CV analysis reporting significant attachment inhibition at 5000 µg/mL (92.81% *p* < 0.0001), 2500 µg/mL (90.70% *p* < 0.0001), 1250 µg/mL (85.15% *p* < 0.0001), 625 µg/mL (79.75% *p* < 0.0001), and 312.5 µg/mL (59.39% *p* < 0.0001), and Rez analysis also reported significant attachment inhibition at the same concentrations of 5000 µg/mL (73.64% *p* = 0.0363), 2500 µg/mL (75.89% *p* = 0.0291), 1250 µg/mL (75.22% *p* = 0.0263), 625 µg/mL (76.13% *p* = 0.0277), and 312.5 µg/mL (79.80% *p* = 0.0354). There were no significant differences observed at other AgNO_3_ concentrations; however, there was an observable increase in biofilm formation and metabolic activity on lid pegs concentrations of 78.13 µg/mL and below.

**Figure 2:**
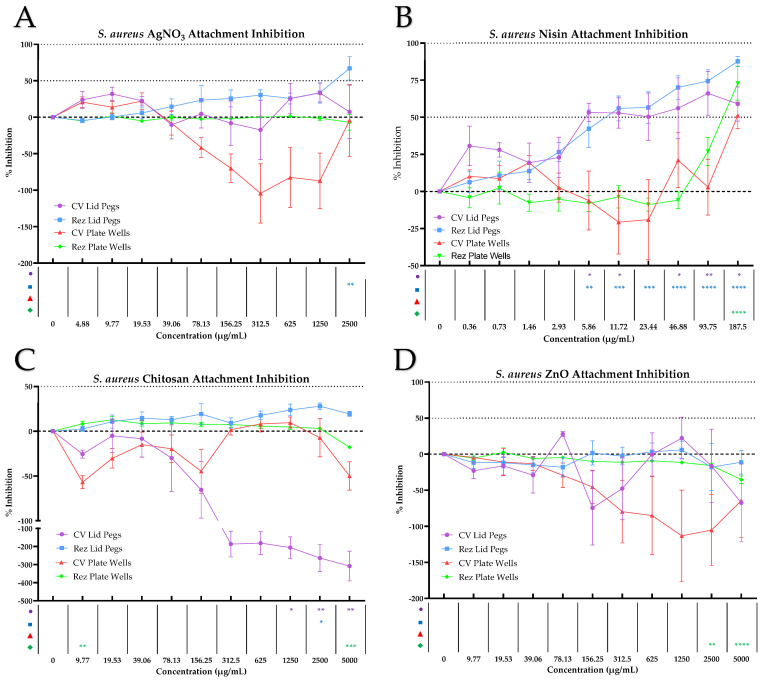
Evaluation of *S. aureus* Attachment Inhibition. Dose-response XY scatter plots presenting eight bioactive and bacteria combinations of (**A**) = AgNO3, (**B**) = Nisin, (**C**) = Chitosan, (**D**) = ZnO. Plots displaying normalised inhibition (%) across a range of concentrations. Inhibition percentages were calculated by normalizing the raw data against the average response of the positive (100% inhibition) and negative (0% inhibition) controls. Data points were fitted with a nonlinear regression model; outliers were identified and removed using the ROUT method (Q = 1%). Significance was determined by use of an ordinary One-Way ANOVA with Dunnett test and is shown by asterisks in GP style (**** *p* < 0.0001, *** *p* < 0.001, ** *p* < 0.01, * *p* < 0.05). Error bars represent the standard error of the mean (SEM) from four independent biological replicates (*n* = 4).

AgNO_3_ was considerably ineffective against *S. aureus* bacterial attachment inhibition on the lid pegs and plate wells (Figure 2A). While CV analysis of the lid pegs did not report any significant responses across AgNO_3_ concentrations of 4.88–2500 µg/mL, Rez analysis presented one significant change, with 67.05% inhibition at 2500 µg/mL (*p* < 0.001). Results from the CV analysis of plate wells did not report significant increases in biofilm formation at any test concentrations 4.88–2500 µg/mL. While there was high variance observed by CV analysis, there was an overall trend of increased biofilm formations at concentrations above 39.06 µg/mL, with the most significant change observed at 312.5 µg/mL (−104.43, *p* = 0.0504). Rez analysis of plate wells did not identify statistically significant results at any concentration between 4.88 and 2500 µg/mL, and it showed negligible inhibition by AgNO_3_ across this range.

#### 3.1.2. Nisin Attachment Inhibition

CV analysis of nisin effects against *P. aeruginosa* did not report significant changes at any concentrations across the range 187.5–0.37 µg/mL (*p* > 0.05). High variability was observed, with inhibitory responses remaining statistically indistinguishable from the control (Figure 1B). Rez analysis of lid pegs showed similar findings, with no significant results (*p* > 0.05) and mean % inhibition values reporting close to 0%. CV analysis of plate wells results presented significant changes versus *P. aeruginosa* at concentrations of 11.72 µg/mL (31.47% *p* = 0.0449) and 1.46 µg/mL (31.96% *p* = 0.0404). Rez analysis of plates did not identify any similar results to that of the CV analysis, and the determined % inhibitions were negligible at all test concentrations.

Nisin exhibited significant inhibition against *S. aureus* at concentrations of 187.5 µg/mL (58.97% *p* = 0.0168), 93.75 µg/mL (66.05% *p* = 0.006), 46.88 µg/mL (56.11% *p* = 0.0251), 11.72 µg/mL (52.90% *p* = 0.0387) and 5.86 µg/mL (53.26% *p* = 0.037), as determined by CV analysis (Figure 2B). There was also inhibition seen at 23.44 µg/mL (50.34%), but it was insignificant in this data set. Rez analysis of the lid pegs also showed significant inhibitions at concentrations of 187.5 µg/mL (87.67% *p* < 0.0001), 93.75 µg/mL (74.44% *p* < 0.0001), 46.88 µg/mL (70.15% *p* < 0.0001), 23.44 µg/mL (56.57% *p* = 0.0002), 11.72 µg/mL (55.97% *p* = 0.0002), and 5.86 µg/mL (42.22% *p* = 0.0055). CV analysis of Nisin against *S. aureus* on the plate wells did not identify any statistically significant responses across the treatment range of 187.5–0.37 µg/mL. While there was inhibition identified at concentrations of 187.5 µg/mL (51.29%), 46.88 µg/mL (21.07%) and 1.46 µg/mL (19.36%), there were also % increases at concentrations of 23.44 µg/mL (−19.07%) and 11.72 µg/mL (−20.69%). Rez analysis of nisin versus *S. aureus* attachment on the plate wells showed significant inhibition (*p* < 0.0001) at 187.5 µg/mL (72.79%). At concentrations <93.75 µg/mL, nisin exhibited negligible changes by Rez analysis.

#### 3.1.3. Chitosan Attachment Inhibition

CV analysis of chitosan against *P. aeruginosa* lid peg biofilms did not report significant changes at any concentration across the range (9.77 – 5000 µg/mL) (Figure 1C). However, lower concentrations of chitosan were noted to cause some inhibition, up to 30.30% at 39.06 µg/mL. Rez analysis also found no significant results, with mean values found to be irrelevant due to high variance. CV attachment analysis on plate wells did not report any significant changes from chitosan exposure to *P. aeruginosa* at all test concentrations (Figure 1C). While not significant, chitosan exhibited varying degrees of attachment inhibition on the plate wells, with CV analysis reporting inhibition at concentrations of 312.50 µg/mL (30.20 ± 6.08% *p* = 0.49), 156.25 µg/mL (27.59 ± 4.06% *p* = 0.5908), 78.13 µg/mL (28.57 ± 4.73% *p* = 0.5525), 39.06 µg/mL (35.94 ± 3.33% *p* = 0.3008), 19.53 µg/mL (23.77 ± 6.61% *p* = 0.7414), and 9.77 µg/mL (18.07 ± 0.99% *p* = 0.9206). Rez analysis found non-significant inhibition at 312.50 µg/mL (25.52 ± 4.15% *p* = 0.798), 156.25 µg/mL (33.10 ± 2.85% *p* = 0.4797), 78.13 µg/mL (23.26 ± 4.58% *p* = 0.8721), 39.06 µg/mL (26.36 ± 1.31% *p* = 0.7666), 19.53 µg/mL (26.10 ± 3.20% *p* = 0.7765), and 9.77 µg/mL (21.04 ± 3.01% *p* = 0.9271), as well as significant attachment increase at 5000 µg/mL (−34.25 ± 16.60% *p* = 0.0152).

*S. aureus* CV analysis found that chitosan exposure during attachment caused significant increases in biofilm formation on the lid pegs at concentrations of 5000 µg/mL (−308.23%, *p* = 0.0012), 2500 µg/mL (−263.98%, *p* = 0.0064) and 1250 µg/mL (−205.98%, *p* = 0.049) (Figure 2C). There were no significant changes below these chitosan concentrations; however, all responses showed a mean increase in biofilm formation. Rez analysis of the lid pegs’ biofilm formation found one significant change at 2500 µg/mL (27.98%, *p* = 0.0184). While non-significant, all other concentrations gave inhibitory responses (Figure 2C). CV analysis of *S. aureus* attachment on the plate wells found no significant changes at all test concentrations. Although some concentrations of chitosan exposure did elicit a notable biofilm growth response (−50.06% at 5000 µg/mL, −44.78% at 156.25 µg/mL, −30.28% at 19.53 µg/mL, −56.96% at 9.77 µg/mL), these were not statistically significant. Rez analysis of the plate wells found a significant inhibition of biofilm activity at 19.53 µg/mL (12.68%, *p* = 0.009) and also a significant increase in biofilm activity at 5000 µg/mL (−17.77%, *p* = 0.0002) (Figure 2C). There were no significant responses at other test concentrations, likely reflecting a minimal effect size as the values were near 0%.

#### 3.1.4. ZnO Attachment Inhibition

ZnO was observed to cause moderate attachment inhibition against *P. aeruginosa* on lid pegs, as determined by CV analysis; however, there were no significant changes (Figure 1D). While inhibition was seen to increase with ZnO concentrations, it did not reach higher than 54.53 ± 11.96% (*p* = 0.9768) at 5000 µg/mL. Rez analysis found no significant changes to biofilm-embedded, metabolically active bacterial cells of the lid peg biofilms and also did not recognize any relevant inhibition, with the results reflecting a minimal effect near 0% with variance (Figure 1D). ZnO against *P. aeruginosa* on the plate wells exhibited no significant changes of attachment by CV analysis. There was low inhibition exhibited at concentrations of 156.25 µg/mL (12.64 ± 9.59% *p* = 0.9877), 78.13 µg/mL (21.95 ± 2.28% *p* = 0.7637), 39.06 µg/mL (16.21 ± 6.26% *p* = 0.9404), 19.53 µg/mL (16.30 ± 2.84% *p* = 0.9385), and 9.77 µg/mL (15.28 ± 2.40% *p* = 0.9573). Rez analysis of the plate wells found no statistically significant changes at all concentrations, with no notable effect versus the *P. aeruginosa* attachment observed (Figure 1D).

There were no significant effects observed against *S. aureus* attachment on the lid pegs, as determined by both CV analysis and Rez analysis; however, there was a noticeable trend of increased biofilm activity seen, although the SEM for these results was high (Figure 2D). While the results from the plate wells showed increases in biofilm formations through CV analysis, they were not significant, which was most likely due to high variance. Rez analysis identified significant increases at concentrations of 5000 µg/mL (−35.40%, *p* < 0.0001) and 25,000 µg/mL (−15.99%, *p* = 0.0058) (Figure 2D).

### 3.2. Biofilm Inhibition

#### 3.2.1. AgNO_3_ Biofilm Inhibition

**Figure 3:**
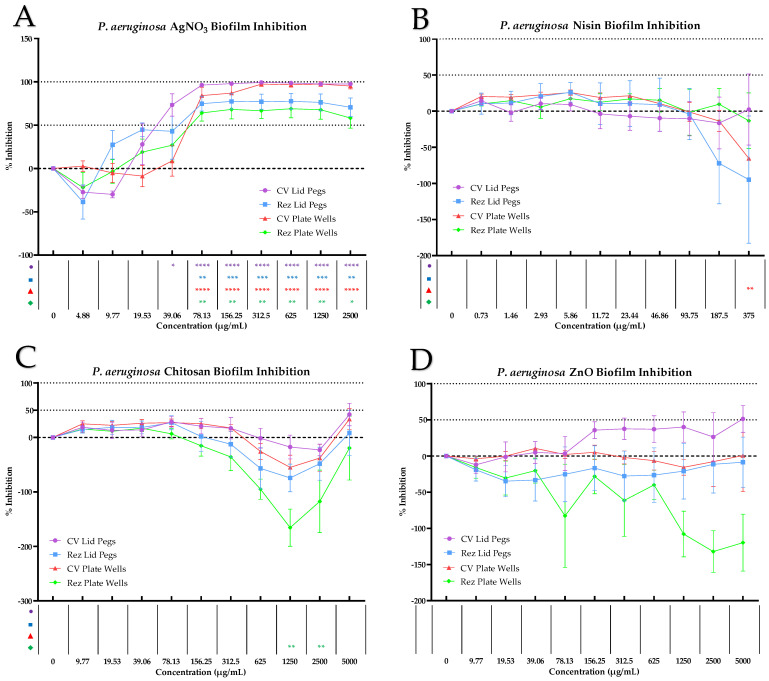
Evaluation of *P. aeruginosa* Biofilm Growth Inhibition. Dose-response XY scatter plots presenting four bioactives, (**A**) = AgNO3, (**B**) = Nisin, (**C**) = Chitosan, (**D**) = ZnO, against *S. aureus* biofilm growth. Plots displaying normalised inhibition (%) across a range of concentrations (µg/mL). Each graph shows effects, as determined by crystal violet (CV) and resazurin (Rez), from biofilm growth on plate wells and lid pegs. Inhibition percentages were calculated by normalising the raw data against the average response of the positive (100% inhibition) and negative (0% inhibition) controls. Data points were fitted with a nonlinear regression model; outliers were identified and removed using the ROUT method (Q = 1%). Significance was determined by use of an ordinary One-Way ANOVA with Dunnett test and is shown by asterisks in GP style (**** *p* < 0.0001, *** *p* < 0.001, ** *p* < 0.01, * *p* < 0.05). Error bars represent the standard error of the mean (SEM) from four independent biological replicates (*n* = 4).

AgNO_3_ exhibited high biofilm inhibition against *P. aeruginosa* on the lid pegs, as shown by both CV analyses (Figure 3A). Statistical analysis found significant inhibition at all concentrations ≥78.125 µg/mL, exhibiting at least 96.29–99.21% inhibition (*p* < 0.0001). There was also significant inhibition at 39.06 µg/mL (45.96%, *p* = 0.0141). Lower concentrations of AgNO_3_ showed non-significant increases in biofilm formation at 9.77 µg/mL (−29.92%, *p* = 0.3191) and 4.88 µg/mL (−27.33%, *p* = 0.212). Rez analysis of the lid pegs showed significant inhibition at concentrations of 2500 µg/mL (70.58%, *p* = 0.0021), 1250 µg/mL (76.28%, *p* = 0.0009), 625 µg/mL (77.47%, *p* = 0.0007), 312.5 µg/mL (77.00%, *p* = 0.0008), 156.25 µg/mL (77.35%, *p* = 0.0007), and 78.13 µg/mL (74.76%, *p* = 0.0011). Similar results were exhibited from AgNO_3_ biofilm inhibition against *P. aeruginosa* on the plate wells (Figure 3A). Statistical analysis of the CV results showed significant inhibition (*p* < 0.0001) at concentrations of 78.13 µg/mL (84.13%), 156.25 µg/mL (86.89%,) 312.5 µg/mL (94.40%), 625 µg/mL (96.65%), 1250 µg/mL (97.37%) and 2500 µg/mL (95.21%). There was significant inhibition shown by the Rez results on the plate wells at concentrations of 2500 µg/mL (58.22%, *p* = 0.0178), 1250 µg/mL (67.71%, *p* = 0.0044), 625 µg/mL (68.85%, *p* = 0.0037), 312.5 µg/mL (66.57%, *p* = 0.0052), 156.25 µg/mL (68.16%, *p* = 0.0041), and 78.13 µg/mL (64.14%, *p* = 0.0075).

**Figure 4:**
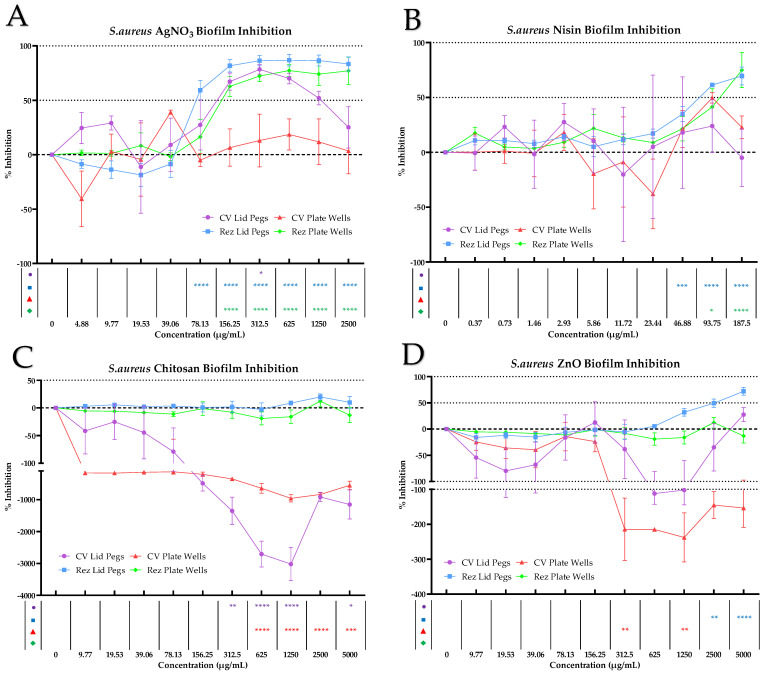
Evaluation of *S. aureus* Biofilm Growth Inhibition. Dose-response XY scatter plots presenting four bioactives, (**A**) = AgNO3, (**B**) = Nisin, (**C**) = Chitosan, (**D**) = ZnO, against *S. aureus* biofilm growth. Plots displaying normalised inhibition (%) across a range of concentrations (µg/mL). Each graph shows effects, as determined by crystal violet (CV) and resazurin (Rez), from biofilm growth on plate wells and lid pegs. Inhibition percentages were calculated by normalising the raw data against the average response of the positive (100% inhibition) and negative (0% inhibition) controls. Data points were fitted with a nonlinear regression model; outliers were identified and removed using the ROUT method (Q = 1%). Significance was determined by use of an ordinary One-Way ANOVA with Dunnett test and is shown by asterisks in GP style (**** *p* < 0.0001, *** *p* < 0.001, ** *p* < 0.01, * *p* < 0.05). Error bars represent the standard error of the mean (SEM) from four independent biological replicates (*n* = 4).

AgNO_3_ exhibited significant inhibition above 312.5 µg/mL (78.48%, *p* = 0.0352) versus *S. aureus* lid peg biofilms, as shown through analysis of the CV results. While non-significant, there was marked inhibition at concentrations of 156.25 µg/mL (67.21%, *p* = 0.094), 625 µg/mL (70.11%, *p* = 0.0737) and 1250 µg/mL (52.03%, *p* = 0.2904) (Figure 4A). The highest concentration 2500 µg/mL exhibited lower inhibition (25.18%) and was also non-significant (*p* = 0.9314). Statistical analysis of the Rez results found significant inhibition (*p* < 0.0001) at concentrations of 2500 µg/mL (83.41%, *p* < 0.0001), 1250 µg/mL (86.52% *p* < 0.0001), 625 µg/mL (86.85% *p* < 0.0001), 312.5 µg/mL (86.38% *p* < 0.0001), 156.25 µg/mL (81.79% *p* < 0.0001), 78.13 µg/mL (59.25% *p* < 0.0001).

Results from *S. aureus* biofilm growth on the plate wells did not report any significant inhibition across test concentrations of 4.88–2500 µg/mL, with the % inhibition values staying close to 0% (Figure 4A). Rez analysis did identify significant changes at concentrations of 156.25–2500 µg/mL (*p* < 0.0001), with results of 2500 µg/mL (77.10% *p* < 0.0001), 1250 µg/mL (74.09% *p* < 0.0001), 625 µg/mL (77.33% *p* < 0.0001), 312.5 µg/mL (72.33% *p* < 0.0001), and 156.25 µg/mL (62.78% *p* < 0.0001).

#### 3.2.2. Nisin Biofilm Inhibition

Nisin did not present any statistically significant results versus *P. aeruginosa* on lid pegs by CV analysis. The determined values varied across the treatment range of 0.37–187.5 µg/mL nisin, with the high SEM and closeness to 0% deeming them biologically insignificant. Similarly, nisin produced no significant changes in biofilm activity, as determined by Rez analysis. While the highest concentrations (187.5 and 93.75 µg/mL) showed a mean increase in metabolic activity (−94.86% and −72.22% inhibition, respectively), these trends were non-significant due to high variability (SEM = 88.15 and 56.13) (Figure 3B).

CV analysis of nisin versus *P. aeruginosa* biofilm growth on the plate wells found one significant value at the concentration 375 µg/mL (−65.30% *p* = 0.0023); however, as this result was based on a single biological replicate following outlier removal (Q = 1%), it was excluded from the final interpretation of nisin’s overall effect. Analysis of the Rez results did not identify any significant changes, and the results did not exhibit any notable effect, giving close to 0% at all concentrations (Figure 3B).

Nisin did not show any significant changes against *S. aureus* on the lid pegs through CV analysis; however, Rez analysis identified significant inhibitions at concentrations 187.5 µg/mL (69.59% *p* < 0.0001), 93.75 µg/mL (61.33% *p* < 0.0001) and 46.88 µg/mL (35.04% *p* = 0.0002) (Figure 4B). Nisin did not demonstrate any notable or significant effects against *S. aureus* on the plate wells by CV analysis, with the mean values being negligible or inconsistent (Figure 4B). However, Rez analysis found significance at concentrations of 93.75 µg/mL (74.96%m *p* < 0.0001) and 187.5 µg/mL (41.38% *p* = 0.0216).

#### 3.2.3. Chitosan Biofilm Inhibition

Chitosan did not report significant effects against *P. aeruginosa* on the lid pegs by CV analysis. While there were some results showing values of 42.08% inhibition at 5000 µg/mL, the variance (SEM 20.58) made this negligible (Figure 3C). Rez analysis of the lid pegs did not identify any significant changes and described negligible mean results due to variance; however, while non-significant, there were noticeable increase in biofilm formation at 2500 µg/mL (−48.11% inhibition SEM 30.25 *p* = 0.6851), 1250 µg/mL (−74.19% inhibition SEM 5.34 *p* = 0.226) and 625 µg/mL (−56.71% inhibition SEM 38.00 *p* = 0.5078).

*P. aeruginosa* biofilm growth on the plate wells did not report any significant effect from chitosan treatment. There were noted increases in biofilm formation at concentrations of 2500 µg/mL (−37.70 ± 25.12% *p* = 0.3092), 1250 µg/mL (−54.97 ± 22.31% *p* = 0.0505), and 625 µg/mL (−25.89 ± 14.51% *p* = 0.7141), but these were most likely non-significant due to the observed variance. Rez analysis of the plate wells found significant increases in biofilm activity at concentrations of 2500 µg/mL (−156.78 ± 56.67% *p* = 0.0048) and 1250 µg/mL (−165.65 ± 34.17% *p* = 0.0026). The results at 625 µg/mL (−95.00 ± 18.55% *p* = 0.1666) were noticeable but regarded non-significant.

Chitosan exhibited increases in biofilm growth against *S. aureus* on the lid pegs at all test concentrations by CV analysis (Figure 4C). There were significant increases seen at 5000 µg/mL (−1151.70% *p* = 0.0186), 1250 µg/mL (−3023.13% *p* < 0.0001), 625 µg/mL (−2708.35% *p* < 0.0001), and 312.5 µg/mL (−1354.79% *p* = 0.0089). There were noteworthy increases at test concentrations of 2500 µg/mL (−918.06% *p* = 0.0877), 312.5 µg/mL (−1354.79% *p* = 0.0089), 156.25 µg/mL (−488.98% *p* = 0.685), 78.13 µg/mL (−79.04% *p* = 0.9997), 39.06 µg/mL (−44.57% *p* = 0.9999), 19.53 µg/mL (−25.31% *p* = >0.9999), and 9.77 µg/mL (−41.59% *p* = 0.9999); however, these were deemed non-significant most likely due to extreme increases seen at higher concentrations,] and the large variance at lower concentrations (relative to the mean). Rez analysis of the lid pegs did not show any significant or noteworthy changes across all test concentrations, with the majority of results returning closeness to zero (with variance accounted).

Chitosan results against *S. aureus* on the plate wells found considerable increases at all concentrations as per CV analysis, with significant increases seen at 5000 µg/mL (−552.88% *p* = 0.0007), 2500 µg/mL (−839.34% *p* < 0.0001), 1250 µg/mL (−960.61% *p* < 0.0001), and 625 µg/mL (−643.71% *p* < 0.0001) (Figure 4C). The remaining results found notable increases at concentrations of 312.5 µg/mL (−347.96% *p* = 0.0535), 156.25 µg/mL (−206.43% *p* = 0.4766), 78.13 µg/mL (−129.48% *p* = 0.8938), 39.06 µg/mL (−140.84% *p* = 0.8448), 19.53 µg/mL (−167.24% *p* = 0.7031), and 9.77 µg/mL (−162.79% *p* = 0.7287). Rez analysis of the plate wells did not report any significant or notable non-significant changes across the test concentration range, with all results approaching zero.

#### 3.2.4. ZnO Biofilm Inhibition

ZnO exhibited biofilm inhibition against *P. aeruginosa* on the lid pegs at the treatment range of 156.25–5000 µg/mL, as identified by CV analysis (Figure 3D). While there were no significant results identified, there were notable differences at concentrations of 5000 µg/mL (51.30% *p* = 0.3399), 2500 µg/mL (26.30% *p* = 0.9279), 1250 µg/mL (40.08% *p* = 0.6128), 625 µg/mL (37.11% *p* = 0.6925), 312.5 µg/mL (37.50% *p* = 0.6821), and 156.25 µg/mL (35.74% *p* = 0.7286). Rez analysis did not show any significant differences across the treatment range. While the means trended toward increased biofilm activity, high variance meant the results were consistent with zero, thus rendering any observed effect negligible.

The results of ZnO versus *S. aureus* biofilm growth on the lid pegs did not report any significant effects through CV analysis, with the results showing mixed effects at different concentrations (Figure 4D). There was inhibition noted at the highest concentration of 5000 µg/mL (27.53%, *p* = 0.9975) and at 156.25 µg/mL (12.72% *p* = 0.9997), with biofilm increases observed at lower concentrations of 2500 µg/mL (−34.97% *p* = 0.9935), 1250 µg/mL (−102.40% *p* = 0.3719), 625 µg/mL (−112.18% *p* = 0.2776), 312.5 µg/mL (−38.47% *p* = 0.9911), 78.13 µg/mL (−16.34% *p* = 0.9996), 39.06 µg/mL (−68.01% *p* = 0.7956), 19.53 µg/mL (−79.77% *p* = 0.6477), and 9.77 µg/mL (−54.52% *p* = 0.9248). Rez analysis of lid peg growth found significant inhibition at higher concentrations of 5000 µg/mL (71.98% *p* < 0.0001) and 2500 µg/mL (49.34% *p* = 0.0015). There was notable inhibition at 1250 µg/mL (31.94% *p* = 0.0669), but it was not considered significant. The mean results at concentrations below 1250 µg/mL were negligible due to variance, remaining close to zero.

Analysis of plates well biofilm growth through CV found significant inhibition at concentrations of 1250 µg/mL (−237.82% *p* = 0.0034) and 312.5 µg/mL (−214.79% *p* = 0.0091). While non-significant, there was notable inhibition at concentrations of 5000 µg/mL (−153.31% *p* = 0.0649) and 2500 µg/mL (−145.18% *p* = 0.1341). The 625 µg/mL concentration exhibited inhibition (−214.81% *p* = 0.1271); however, this result was based on a single biological replicate following outlier removal (Q = 1%) and so was not considered in the final conclusions. Rez analysis of the plate wells found no significant or noteworthy results, with all concentrations results remaining close to zero.

### 3.3. Biofilm Reduction

#### 3.3.1. AgNO_3_ Biofilm Reduction

**Figure 5:**
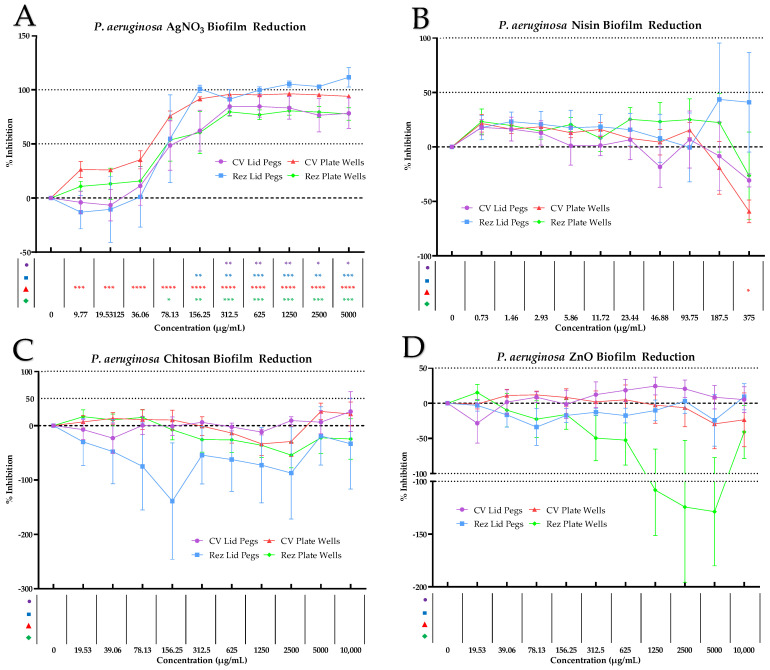
Evaluation of *P. aeruginosa* Biofilm Reduction. Dose-response XY scatter plots presenting four bioactives, (**A**) = AgNO3, (**B**) = Nisin, (**C**) = Chitosan, (**D**) = ZnO, against established *P. aeruginosa* biofilms. Plots displaying normalised reduction (%) across a range of concentrations (µg/mL). Each graph shows effects, as determined by crystal violet (CV) and resazurin (Rez), from biofilms grown on plate wells and lid pegs. Reduction percentages were calculated by normalising the raw data against the average response of the positive (100% reduction) and negative (0% reduction) controls. Data points were fitted with a nonlinear regression model; outliers were identified and removed using the ROUT method (Q = 1%). Significance was determined by use of an ordinary One-Way ANOVA with Dunnett test and is shown by asterisks in GP style (**** *p* < 0.0001, *** *p* < 0.001, ** *p* < 0.01, * *p* < 0.05). Error bars represent the standard error of the mean (SEM) from four independent biological replicates (*n* = 4).

Results from the CV analysis of AgNO_3_ versus *P. aeruginosa* biofilms grown on lid pegs showed significant reductions at concentrations of 5000 µg/mL (78.26 ± 10.96% *p* = 0.0175), 2500 µg/mL (76.42 ± 11.94% *p* = 0.0213), 1250 µg/mL (83.21 ± 10.43% *p* = 0.0049), 625 µg/mL (84.51 ± 19.76% *p* = 0.0042), and 312.5 µg/mL (84.65 ± 25.19% *p* = 0.0041) (Figure 5A). There was notable inhibition at concentrations 156.25 µg/mL (62.27 ± 10.61% *p* = 0.0534) and 78.13 µg/mL (43.82 ± 27.39% *p* = 0.3267); however, they were considered non-significant by One-Way ANOVA analysis. Rez analysis of the lid peg biofilms found significant inhibition at concentrations of 5000 µg/mL (111.57 ± 9.01% *p* = 0.0006), 2500 µg/mL (102.92 ± 1.48% *p* = 0.0015), 1250 µg/mL (105.15 ± 2.93% *p* = 0.0005), 625 µg/mL (99.69 ± 3.04% *p* = 0.0009), 312.5 µg/mL (91.20 ± 9.04% *p* = 0.0024), and 156.25 µg/mL (100.82 ± 3.37% *p* = 0.0066). The mean results at other treatment concentrations were considered negligible due to closeness to zero or negating variance. Results from the CV analysis of biofilm growth on the plates wells found significant inhibition at all test concentrations, 5000 µg/mL (93.97 ± 1.24% *p* < 0.0001), 2500 µg/mL (95.20 ± 0.91% *p* < 0.0001), 1250 µg/mL (96.25 ± 1.27% *p* < 0.0001), 625 µg/mL (95.26 ± 1.05% *p* < 0.0001), 312.5 µg/mL (95.79 ± 1.21% *p* < 0.0001), 156.25 µg/mL (91.57 ± 1.92% *p* < 0.0001), 78.13 µg/mL (75.82 ± 4.60% *p* < 0.0001), 39.06 µg/mL (35.32 ± 8.40% *p* < 0.0001), 19.53 µg/mL (25.91 ± 0.51% *p* = 0.0005), and 9.77 µg/mL (26.27 ± 7.54% *p* = 0.0001). Rez analysis of the plate wells found significant inhibition at concentrations of 5000 µg/mL (77.56 ± 5.88% *p* = 0.0005), 2500 µg/mL (79.33 ± 5.02% *p* = 0.0004), 1250 µg/mL (80.47 ± 3.92% *p* = 0.0001), 625 µg/mL (76.90 ± 4.50% *p* = 0.0002), 312.5 µg/mL (79.50 ± 3.92% *p* = 0.0001), 156.25 µg/mL (60.53 ± 19.52% *p* = 0.0036), and 78.13 µg/mL (53.58 ± 19.53% *p* = 0.0116). Lower concentrations exhibited negligible inhibition.

**Figure 6:**
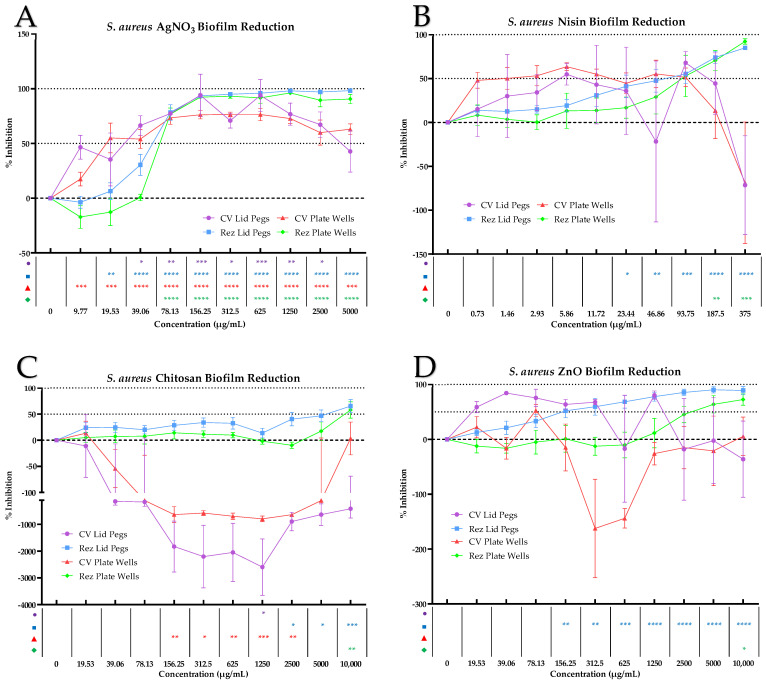
Evaluation of *S. aureus* Biofilm Reduction. Dose-response XY scatter plots presenting four bioactives, (**A**) = AgNO3, (**B**) = Nisin, (**C**) = Chitosan, (**D**) = ZnO, against established *S. aureus* biofilms. Plots displaying normalised reduction (%) across a range of concentrations (µg/mL). Each graph shows effects, as determined by crystal violet (CV) and resazurin (Rez), from biofilms grown on plate wells and lid pegs. Reduction percentages were calculated by normalising the raw data against the average response of the positive (100% reduction) and negative (0% reduction) controls. Data points were fitted with a nonlinear regression model; outliers were identified and removed using the ROUT method (Q = 1%). Error bars represent the standard error of the mean (SEM) from four independent biological replicates (*n* = 4). (**** *p* < 0.0001, *** *p* < 0.001, ** *p* < 0.01, * *p* < 0.05).

CV analysis of the AgNO_3_ results versus *S. aureus* on the lid pegs found significant inhibition at concentrations of 2500 µg/mL (67.15 ± 11.56% *p* = 0.0161), 1250 µg/mL (76.76 ± 10.15% *p* = 0.0052), 625 µg/mL (93.90 ± 14.61% *p* = 0.0007), 312.5 µg/mL (70.80 ± 6.77% *p* = 0.0105), 156.25 µg/mL (94.03 ± 19.24% *p* = 0.0006), 78.13 µg/mL (77.33 ± 1.81% *p* = 0.0048), and 39.06 µg/mL (66.39 ± 8.97% *p* = 0.0175) (Figure 6A). There was inhibition shown at other test concentrations; however, they were below 50% inhibition and non-significant. Rez analysis of the lid pegs found significant inhibition at concentrations of 5000 µg/mL (98.18 ± 0.37% *p* < 0.0001), 2500 µg/mL (97.02 ± 0.52% *p* < 0.0001), 1250 µg/mL (98.04 ± 0.53% *p* < 0.0001), 625 µg/mL (95.90 ± 1.11% *p* < 0.0001), 312.5 µg/mL (94.99 ± 1.50% *p* < 0.0001), 156.25 µg/mL (93.47 ± 1.77% *p* < 0.0001), 78.13 µg/mL (78.36 ± 7.15% *p* < 0.0001), and 39.06 µg/mL (30.47 ± 9.60% *p* = 0.0011). Lower concentrations exhibited effects close to zero.

Results from the plate wells exhibited significant inhibition at concentrations of 5000 µg/mL (62.95 ± 4.98% *p* = 0.0002), 2500 µg/mL (59.95 ± 11.50% *p* < 0.0001), 1250 µg/mL (72.67 ± 4.59% *p* < 0.0001),625 µg/mL (76.41 ± 5.68% *p* < 0.0001), 312.5 µg/mL (76.44 ± 2.21% *p* < 0.0001), 156.25 µg/mL (76.17 ± 3.75% *p* < 0.0001), 78.13 µg/mL (73.41 ± 5.92% *p* < 0.0001), 39.06 µg/mL (53.99 ± 8.73% *p* = 0.0003), and 19.53 µg/mL (55.01 ± 13.57% *p* = 0.0002). Rez analysis found significant inhibition at 5000 µg/L (90.60 ± 4.23% *p* < 0.0001), 2500 µg/mL (89.48 ± 6.01% *p* < 0.0001), 1250 µg/mL (96.13 ± 1.40% *p* < 0.0001), 625 µg/mL (91.63 ± 4.86% *p* < 0.0001), 312.5 µg/mL (93.17 ± 1.89% *p* < 0.0001), 156.25 µg/mL (92.27 ± 1.03% *p* < 0.0001), and 78.13 µg/mL (76.82 ± 5.47% *p* < 0.0001).

#### 3.3.2. Nisin Biofilm Reduction

Nisin had no significant effect versus *P. aeruginosa* on the lid pegs, as determined by CV analysis. There was a notable yet non-significant increase in biofilm formation seen at 375 µg/mL (−30.73 ± 5.97% *p* = 0.8288), while all other treatment concentrations exhibited low mean inhibitory effects with high variance and thus were considered biologically irrelevant (Figure 5B). Rez analysis showed somewhat notable inhibition at the upper concentrations 375 µg/mL (41.00 ± 45.71% *p* = 0.7691) and 187.5 µg/mL (43.56 ± 51.84% *p* = 0.7141); however, these were not considered significant, most likely to high variance. Other test concentrations exhibited mean results close to zero and/or with high variance. CV analysis of nisin versus *P. aeruginosa* on the plate wells found a significant increase in biofilm formation at 375 µg/mL (−59.18 ± 10.42% P = 0.0123). The results at all other test concentrations were considered biologically irrelevant due to their low effect and negating variance. Rez analysis of the plate wells did not report significant effects at any test concertation, with the results showing effects close to zero due to biological variance.

Nisin did not report any significant effect versus *S. aureus* biofilms grown on lid pegs through CV analysis; however, there were notable reports of inhibition at test concentrations of 187.5 (44.24 ± 34.94% *p* = 0.9926), 93.75 (67.84 ± 12.75% *p* = 0.9162), 46.88 (−21.79 ± 91.64% *p* = 0.9995), 23.44 (35.88 ± 49.72% *p* = 0.9966), 11.72 (43.00 ± 44.74% *p* = 0.9914), and 5.86 (54.71 ± 12.20% *p* = 0.9606) and also a notable increase at 375 (−71.42 ± 56.18% *p* = 0.8923) (Figure 6B). The results at other concentrations were not considered due to their low effect or high variance. Rez analysis of the lid peg biofilms showed significant inhibition at concentrations of 375 (84.90 ± 2.18% *p* < 0.0001), 187.5 (73.92 ± 7.01% *p* < 0.0001), 93.75 (55.20 ± 10.97% *p* = 0.0004), 46.88 (47.48 ± 12.99% *p* = 0.0026), and 23.44 (41.07 ± 12.72% *p* = 0.0113). CV analysis of biofilm growth on the plate wells did not report any significant effects across the test concentrations. While non-significant, there were noticeable inhibitory effects at concentrations of 93.75 (51.37 ± 10.57% *p* = 0.6306), 46.88 (55.26 ± 15.69% *p* = 0.5508), 23.44 (44.60 ± 11.82% *p* = 0.7669), 11.72 (54.94 ± 5.76% *p* = 0.5571), 5.86 (63.49 ± 4.48% *p* = 0.3953), 2.93 (53.11 ± 11.47% *p* = 0.5947), 1.46 (50.12 ± 12.34% *p* = 0.6562), and 0.73 (47.89 ± 8.99% *p* = 0.7017). Rez analysis showed significant effects at concentrations of 375 (92.15 ± 3.10% *p* = 0.0004) and 187.5 (70.45 ± 11.38% *p* = 0.0085). There were no other notable effects observed, with the results reporting near zero due to low effect or variance.

#### 3.3.3. Chitosan Biofilm Reduction

Chitosan did not report any significant changes against *P. aeruginosa* biofilms grown on lid pegs through CV analysis across the test concentration range (19.53–10,000 µg/mL, *p* > 0.05) (Figure 5C). While there was both a notable inhibitory effect and stimulatory effect shown, the SEM rendered these results negligible at concentrations 10,000 µg/mL (25.91 ± 36.71% *p* = 0.9302) and 39.06 µg/mL (−22.94 ± 27.88% *p* = 0.9454). All other results remained close to zero. Rez analysis did not report any significant results from chitosan treatment of *P. aeruginosa* biofilms grown on lid pegs (*p* > 0.05). While there was a mean increase see at all test concentrations (10,000–19.53 µg/mL), with some reports of high increases (156.25 µg/mL, −138.77 ± 107.18% *p* = 0.6145), the high biological variance caused these to be found non-significant. CV analysis of chitosan treated biofilms on the plate wells did not report any significant effects across the test concentrations (10,000–19.53 µg/mL, *p* > 0.05) (Figure 5C). Rez analysis also found no significant effect across the test range (10,000–19.53 µg/mL, *p* > 0.05). There was a mean stimulatory effect exhibited at concentrations 10,000–156.25 µg/mL, with 2500 µg/mL having a notable effect (−54.36 ± 23.47% *p* = 0.4275).

CV analysis of chitosan treatment versus *S. aureus* biofilms on the lid pegs showed increases in biofilm formations across all test concentrations (10,000–19.53 µg/mL), with a significant increase observed at 1250 (−2595.83 ± 1053.06% *p* = 0.0316), and large, non-significant increases at the concentrations 10,000 (−417.75 ± 348.84% *p* = 0.9971), 5000 (−637.74 ± 404.60% *p* = 0.9859), 2500 (−893.17 ± 347.55% *p* = 0.8973), 625 (−2048.40 ± 1087.58% *p* = 0.2303), 312.5 (−2204.01 ± 1172.20% *p* = 0.0902), and 156.25 (−1829.25 ± 946.23% *p* = 0.1573) (Figure 6C). While these results showed dramatic changes, they were still non-significant, most likely due to the biological variability observed. Rez analysis of these treatments showed inhibitory effects across the concentration range (10,000–19.53 µg/mL), with significant changes observed at 10,000 (65.41 ± 12.42% *p* = 0.0005), 5000 (46.68 ± 11.18% *p* = 0.0164), and 2500 (40.43 ± 12.84% *p* = 0.0484). There was also notable inhibition exhibited at concentrations of 625 (32.42 ± 11.33% *p* = 0.1643), 312.5 (33.74 ± 8.82% *p* = 0.1363), 156.25 (28.55 ± 9.41% *p* = 0.2737), 78.13 (20.15 ± 7.94% *p* = 0.6511), 39.06 (24.61 ± 9.70% *p* = 0.4306), and 19.53 (23.92 ± 9.81% *p* = 0.4623). Results from CV analysis of the plate well *S. aureus* biofilms found increases in biofilm formations at the treatment range of 5000–39.06 µg/mL, with significant changes at 2500 (−640.29 ± 63.04% *p* = 0.0091), 1250 (−795.28 ± 102.24% *p* = 0.0009), 625 (−696.57 ± 116.58% *p* = 0.004), 312.5 (−579.49 ± 92.01% *p* = 0.0214), and 156.25 (−635.72 ± 298.23% *p* = 0.0047) (Figure 6C). There were notable increases also at 2500 (−112.58 ± 116.76% *p* = 0.992), 39.06 (−107.68 ± 78.78% *p* = 0.993), and 19.53 (−54.33 ± 36.58% *p* = 0.9996); however, these results were non-significant and negligible due to variance. Rez analysis of the plate wells did not report any significant or marked effect at concentrations of 5000–19.53 µg/mL but showed significant inhibition at 10,000 µg/mL (57.93 ± 15.77% *p* = 0.0055).

#### 3.3.4. ZnO Biofilm Reduction

Treatment of *P. aeruginosa* biofilms on lid pegs with ZnO did not report any significant changes to the biofilm mass by CV analysis at concentrations of 10,000–19.53 µg/mL (Figure 5D). There was minor inhibition at concentrations of 2500 µg/mL (20.42 ± 12.65% *p* = 0.9644) and 1250 µg/mL (24.36 ± 12.67% *p* = 0.907), as well as stimulation at 19.53 µg/mL (−28.36 ± 28.35% *p* = 0.8162). Rez analysis did not report any significant changes at the test concentrations (10,000–19.53 µg/mL) but showed a notable increase at 78.13 µg/mL (−33.88 ± 26.21% *p* = 0.6526). CV analysis of biofilm growth on the plate wells did not report any significant or notable effect at concentrations of 10,000–19.53 µg/mL. Rez analysis of the plate well biofilms did not find any significant changes at concentrations of 1000–19.53 µg/mL but showed a notable increases at concentrations of 5000 µg/mL (−128.72 ± 51.29% *p* = 0.0793), 2500 µg/mL (−124.38 ± 71.49% *p* = 0.143), and 1250 µg/mL (−108.32 ± 43.11% *p* = 0.1887).

Analysis of the ZnO treatment versus *S. aureus* on the lid pegs found no significant effects by CV analysis across all the test concentrations of 10,000–19.53 µg/mL, although there were results of notable inhibition at 1250 (80.31 ± 7.86% *p* = 0.9499), 312.5 (67.73 ± 4.66% *p* = 0.9827), 156.25 (63.52 ± 9.06% *p* = 0.981), 78.13 (75.62 ± 15.88% *p* = 0.945), 39.06 (84.23 ± 0.32% *p* = 0.9682), and 19.53 (58.74 ± 10.32% *p* = 0.9922); however, these results were most likely non-significant due to the range of results across the treatment range (411.1% to −308.2% inhibition) (Figure 6D). Rez analysis of the lid pegs showed significant inhibition at concentrations of 10,000 (89.01 ± 7.28% *p* < 0.0001), 5000 (90.30 ± 5.84% *p* < 0.0001), 2500 (85.43 ± 5.84% *p* < 0.0001), 1250 (77.79 ± 8.12% *p* < 0.0001), 625 µg/mL (68.37 ± 11.48% *p* = 0.0002), 312.5 (59.36 ± 15.52% *p* = 0.0012), and 156.25 (51.94 ± 12.57% *p* = 0.0053). There was notable inhibition at lower concentrations of 78.13 (33.14 ± 11.95% *p* = 0.1375), 39.06 (21.01 ± 12.17% *p* = 0.5891), and 19.53 (12.60 ± 5.88% *p* = 0.9503). CV analysis of the plate wells did not report any significant effects from ZnO versus *S. aureus* at test concentrations of 10,000–19.53 µg/mL; however, there were results of notable stimulation at 625 (−143.92 ± 17.77% *p* = 0.1036) and 312.5 (−162.31 ± 89.79% *p* = 0.0507). Rez analysis found significant inhibition at 10,000 (72.44 ± 10.37% *p* = 0.046), and there was also notable inhibition at concentrations of 5000 (63.76 ± 15.74% *p* = 0.0998) and 2500 (45.31 ± 15.03% *p* = 0.3921).

## 4. Discussion

Through use of the methods and procedures described in this study, the total anti-biofilm capabilities of each bioactive compound were assessed against the proven bacterial biofilm species *S. aureus* and *P. aeruginosa*. These findings provided an important insight into the activity of test bioactives and into the behaviour of bacterial populations within biofilms. Additionally, the findings elucidated information on biofilm structures and the differences between the two Gram species’ responses to treatment. A summation of key biofilm mass and biofilm embedded bacteria findings based on the use of these four tested bioactives against *P. aeruginosa* and *S. aureus* is provided in Table 1 and Table 2, respectively.

### 4.1. Crystal Violet Analysis vs. Resazurin Analysis

The use of both CV and Rez analysis has revealed numerous interactions and insights for how either *P. aeruginosa* or *S. aureus* react to the effects of the bioactives and how their behaviour can differ depending on either a lid peg or plate well growth surface. A noted drawback in the use of Rez analysis is the differing reduction rates between bacterial species. It was noted that *S. aureus* converted resazurin to resorufin at a much higher rate than *P. aeruginosa*, which restricted the establishment of a standardised analysis protocol. Readings for analysis were taken from *S. aureus* at the 2-h time points and at the 4-h time points for *P. aeruginosa*. Through hourly measurements as the standard, it was possible to identify the most suitable timepoint and use the results from those times.

One of the initial observations of interest is how the Rez assay appears to exhibit an upper limitation to reported bacterial attachment and biofilm growth inhibition. This is particularly seen in assays using AgNO_3_, as this was shown to exhibit superior treatment and was corroborated by other studies to be effective [46]. In attachment inhibition assays against *P. aeruginosa*, the CV results show marked inhibition at higher concentrations, up to 99.29% on lid pegs and 92.81% on plate wells (Figure 1A). However, albeit also effective, a side-by-side comparison of Rez indicator data reveals that the same concentration of AgNO_3_ peaks at 83.03% on lid pegs and 79.80% on plate wells. Similar results were shown from biofilm inhibition of *P. aeruginosa*, with inhibition ranging from 96.29 to 99.21% on lid pegs and 84.13 to 97.40% on plate wells through CV analysis, and Rez analysis reporting 70.58 to 77.47% on lid pegs and 58.22 to 64.14% on plate wells at the same concentrations of AgNO_3_.

These results intimate that there was complete inhibition of *P. aeruginosa* bacterial attachment, as determined by CV analysis. However, the use of Rez analysis showed that there were still metabolically active bacteria present. The CV analysis found a reduction range of 56.13–83.36% on lid pegs, whereas the Rez analysis showed reductions of 88.61–111.98% at the same concentration range (5000–156.25 µg/mL) (Figure 5A). However, biofilm reduction results on plate wells exhibited a similar trend to that of the other assays, with CV analysis reporting 81.67–96.17% reduction and Rez analysis reporting 58.46–80.21% on plate wells at 5000–156.25 µg/mL.

Results from the AgNO_3_ treatment of *S. aureus* were surprising; however, other published studies have noted that silver requires extended exposure in order to exhibit full inhibitory effect [40]. Although the use of AgNO_3_ did not exhibit marked effects against *S. aureus*, there were indications that the biofilm mass and embedded bacterial activity do not always correlate with one another (Figure 2A). During attachment inhibition, it was shown that AgNO_3_ caused quite varied effects against the biofilm mass on lid pegs, whereas the biofilm embedded bacterial activity on lid pegs was shown to decrease in a dose-dependent manner. Similarly, in plate wells, AgNO_3_ exhibited notable increases to biofilm formations, which were not reflected in the Rez analysis results, which showed negligible effects at all concentrations. Such occurrence was also noted in biofilm inhibition assays, with lid pegs reporting higher inhibitory effect against biofilm embedded bacterial cells than that of the outer biofilm mass, even though there was significant reductions of biofilm mass shown.

Nisin was found to be somewhat ineffective against *P. aeruginosa*; however, this was expected due to the nature of the bioactive and its inability to affect Gram-negative bacteria. Nisin’s mechanism of action involves binding of the intramembrane bound molecule lipid-II, which is only possible against Gram-positive bacteria which lack an outer membrane [47]. However, effects against Gram-negative biofilm-forming bacteria are not well documented, nor are effects against the biofilm formations. Moreover, some notable effects using nisin are shown in Figure 1B and Figure 5B. CV analysis of the attachment inhibition on plate wells found significant inhibition at concentrations of 11.72 µg/mL (31.47% *p* = 0.0449) and 1.46 µg/mL (31.96%, *p* = 0.0404), as well as nominal inhibition at other concentrations in the range of 93.75–0.37 µg/mL (23.92–30.23%), while Rez analysis did not report any notable findings, with most treatments returning near-zero results (Figure 1B). Such findings show that nisin can affect the external biofilm formation of *P. aeruginosa*. Nisin is reported to bind with the lipid-II molecule, forming pores or holes in the bacteria cellular membrane, suggesting that lipid-II is implicated in the biofilm formation [48,49]. Further studies are merited on this topic. Results from *S. aureus* assays exhibited the inverse, wherein Rez analysis revealed a greater effect upon biofilm embedded bacteria by use of nisin than against the external biofilm mass itself. However, this was not unexpected, as nisin is capable of interacting with the lipid-II of *S. aureus* cells. Throughout the attachment inhibition assays, data generated from the CV and Rez studies closely matched each other.

Similar observations occurred during the biofilm inhibition assay using plate wells and for the biofilm reduction assay, where there was a significant increase in biofilm formation of *P. aeruginosa* at 375 µg/mL (−59.18%, *p* = 0.0123). However, there was also an increase shown from Rez analysis at the same concentration (−26.43%). Conversely, the lid peg analysis from the biofilm inhibition demonstrated quite the opposite, with CV analysis of lid pegs showing somewhat negligible effects, while Rez analysis found increases in biofilm-embedded bacteria activity at concentrations of 187.5 µg/mL (CV: 2.31%, Rez: −94.86%) and 93.75 µg/mL (CV: −16.49%, Rez:−72.22%). These results highlight an unexpected response from *P. aeruginosa* biofilms on lid pegs compared to that on plate wells. There have been reports that compounds with functional groups such as amines and amides can lead to interference with Rez analysis by causing non-enzymatic reduction of resazurin to resorufin, giving false positives, which may explain these increases; however, the same occurrences were not observed across assays or with *S. aureus* after nisin exposure [50]. The results from *S. aureus* exhibited a more stable dose-dependent response against biofilm-embedded bacterial activity, whereas the effects against biofilm mass were varied and did not correlate with the Rez results. This varied effect against the biofilm mass may result from the natural variation of *S. aureus* biofilm matrix composition [51]. Previous studies have exhibited that sub-MIC levels of nisin were seen to upregulate expression of genes associated with bacterial adhesion and polymer matrix production, which may explain biofilm mass increases [52]. The greater effect of nisin against *S. aureus* viability within the biofilm has also been exhibited by researchers in other published studies [10].

Chitosan exhibited varied effects upon *P. aeruginosa* biofilms, which depended on the stage of treatment application and surface. Moreover, differences were much more prominent by comparing findings from use of the CV and Rez stains. While attachment assays against *P. aeruginosa* did not show significant changes on lid pegs, minor effects were reported when using both CV and Rez stains. A significant inhibitory effect was exhibited on plate wells using Rez (5000 µg/mL, −34.25%, *p* = 0.0152) that was somewhat comparable to the CV result (−8.41%). Low concentrations of chitosan promoted inhibition of *P. aeruginosa* biofilms. Increasing concentrations of chitosan caused significant changes in biofilm mass when using *S. aureus* that were observed for attachment inhibition, biofilm inhibition and biofilm reduction (Figure 2C, Figure 4C and Figure 6C). While there were significant increases in *S. aureus* biofilms observed during attachment inhibition assays, a reduction was measured in biofilm embedded bacterial activity that differed from the CV assay. Interestingly, 5000 µg/mL chitosan was the only concentration seen to increase bacterial metabolic activity, as evident on plate wells. Previous studies reported that *P. aeruginosa* will potentially produce more biofilm in response to external stressors, such as antibiotics, which would also support this observation [53]. Chitosan was shown to increase biofilm formation, along with increased bacterial activities at different concentrations (Figure 3C).

Analysis of the biofilm reduction assay yielded interesting results, wherein the biofilm mass on lid pegs and plate wells were unaffected by chitosan across the test concentrations, and the bacteria metabolic activity was increased quite noticeably, as determined by Rez analysis (Figure 5C). *S. aureus* biofilm reduction assay showed substantial increases in biofilm mass, with peaks at 1250 µg/mL, while there was significant inhibition of bacterial activity (Figure 6C). The observed difference in responses were relevant, given that analysis solely through the CV assay would have indicated chitosan to be a stimulant, rather than inhibitor, whereas using Rez analysis alone would have indicated chitosan to be a successful treatment of biofilms. It was noted that biofilm-embedded metabolically active cells were inhibited by chitosan, but this did not influence the external biofilm mass. These observations do not corroborate previous published studies, where chitosan coatings reduced biofilm formations for different bacterial species, including *P. aeruginosa* and *S. aureus* [54]. These studies used alternative forms of chitosan that are worth further consideration [55].

The use of ZnO pre-treatment against *P. aeruginosa* on lid pegs revealed interesting results, where it inhibited biofilm attachment, but this was only evident when using CV stain. Commensurately, there were some observed increases in bacterial activity at concentrations below 1250 µg/mL using ZnO. Conversely, when using plate wells, ZnO was seen to cause inhibition at the lowest concentration of 9.77 µg/mL, which then decreased with concentration, showing a concentration-dependent decline in apparent inhibition. However, use of Rez analysis produced no noteworthy changes. These results suggest that ZnO pre-treatment was disrupting biofilm formation, but not the embedded bacteria’s metabolic activity. The slight increase on lid pegs at the mid-range concentration may indicate, similarly to chitosan, a stress induced elevation of bacteria activity where higher concentrations were potent enough to being inhibiting, and perhaps a higher concentration of ZnO would yield even greater inhibition. *S. aureus* also presented interesting results, where Rez analysis showed that ZnO was ineffective at promoting biofilm attachment on lid pegs, but increased attachment on plate wells occurred (Figure 2D).

*P. aeruginosa* ZnO biofilm inhibition assays presented comparable results to attachment inhibition, with biofilm mass reduction at higher concentrations on lid pegs and an increase in bacterial activity. Plate well analysis revealed interesting results, with the external biofilm mass remaining mostly unaffected at all test concentrations, while biofilm-embedded bacteria metabolic activity increased. Previous studies analysing ZnO effects versus *P. aeruginosa* biofilms found it to hold very effective biofilm inhibition against those grow on plate well walls; however, these studies produced their own ZnO-NPs, which may hold much different effects that the ZnO used presently [34]. *S. aureus* results show a marked increase in biofilm mass on lid pegs and plate wells, with both rising to a peak at 1250 µg/mL and then lowering at higher concentrations. It was found that the biofilm-embedded bacterial populations on lid pegs were significantly affected, with growth inhibition of up to 71.98% (*p* < 0.0001).

Biofilm reduction of *P. aeruginosa* by the use of ZnO also demonstrated similar effects and trends to biofilm inhibition, with lid pegs showing minor biofilm mass reduction and the internally embedded bacterial showing minor increases in activity. The plate wells were found to exhibit very minor reduction (close to zero) at lower concentrations, which then moved into increases at higher concentrations. The Rez analysis revealed a similar trend; however, only the lowest concentration caused reduction, with increases seen at all other concentrations, peaking at 5000 µg/mL and then reducing at 10,000 µg/mL. *S. aureus* biofilm reduction also presented similar results to biofilm inhibition, except for the fact the ZnO exhibited significant biofilm-embedded bacterial cell reduction on both lid pegs and plate wells. ZnO’s inability to affect bacterial cell populations in *P. aeruginosa* may be due the bacteria’s physical changes upon initiating biofilm development. ZnO’s main mechanism of action involves interacting with bacterial cell membranes, causing ruptures in the outer layers. A number of previous studies have noted a large shift in a bacteria’s genetic and physical behaviour upon initiating biofilm development, with one such change being the loss of a solid, membrane form [56]. This loss of a cellular membrane may disable ZnO from interacting with the actual bacterial cells, which may explain effects against *P. aeruginosa* but not those against *S. aureus*. There have been reports of nanoparticles with photocatalytic activity interfering with Rez readings, causing degradation of the dye leading to overestimation of reduced conversion rates, which may explain these results exhibited by *S. aureus*; however, the same occurrence is not observed across other assays of ZnO, and would also be expected by *P. aeruginosa* [57].

### 4.2. Growth Surfaces and Bacterial Type

The use of 96-well plates and lid pegs have been well documented in anti-biofilm assays; however, the use of both surfaces as a combinational experimental design has yet to be reported. By utilizing both in this study, it was possible to show how bacteria as well as antimicrobial treatments will differ in their activities depending on the growth surface. This was coupled with using anti-biofilm assessments (attachment inhibition, biofilm inhibition, biofilm reduction) and both CV and Rez stain analysis, which allows extensive insight into mechanistic ant-biofilm activities. Additionally, *P. aeruginosa* is a motile pathogen that can introduce complexity into biofilm studies. For example, as the lid pegs are essentially suspended in the growth media, it would be assumed that the bacterial cell would require additional means to reach it in order to attach. However, while *S. aureus* is technically non-motile, it is described to move due to Brownian motion which allows this initial contact with the peg lids [58]. Furthermore, as previously published, *S. aureus* expresses Microbial Surface Components Recognizing Adhesive Matrix Molecules (MSCRAMMs) which enables much higher-biding affinity during initial attachment [59]. Results from lid pegs and plate wells have shown *S. aureus* to form quite large biofilms on both surfaces. It was found that plate well biofilms exhibited a higher degree of resilience to treatments, with the exception of chitosan which exhibited much higher increases of biofilm mass on lid pegs. While there is the risk that bacteria may adhere to the bottom of the plate wells, particularly non-motile bacteria, and form dense, almost passive biofilms, the initial 1-h attachment time utilized in this study aims to avoid such occurrences. The findings support *S. aureus*’ affinity to form more of a passive biofilm rather than active as it relies on passive forces for movement [60,61]. In contrast, *P. aeruginosa*, being a motile bacteria, can actively seek out surfaces for attachment and biofilm formation [62]. It was also reported that due to its active motility, biofilm formations can be more irregular and elevated from the surface due to bacterial cell movement on the surfaces post-attachment. These differences in attachment and biofilm structural shape could give explanation to the different response to treatments observed.

Due to *P. aeruginosa*’s active motility, the species is less reliant on the strength of initial attachment and even relies on a weaker affinity to allow bacterial cells to migrate post-attachment, whereas *S. aureus* is highly dependent on its strong binding affinity to enable initial attachment [59]. The differences in these concepts can be observed from AgNO_3_ treatment at the attachment phase (Figure 1A and Figure 2A). AgNO_3_ has been previously reported to be highly effective antimicrobial against both *S. aureus* and *P. aeruginosa*, and this is evident from other results in the present study; however, following exposure at the attachment phase, *S. aureus* exhibited minor inhibitory effects, even showing increases at certain concentrations, whereas *P. aeruginosa* was significantly reduced [25]. Similar results can be seen from chitosan and ZnO exposure, with *S. aureus* attachment increasing across concentrations (Figure 2C,D). While it has been reported that *P. aeruginosa* increases biofilm-related gene regulation following exposure to external stressors, these results indicate a similar response by *S. aureus*, particularly with respect to attachment engagement. As previously mentioned, there is a trend across all treatments and assays where *P. aeruginosa* external biofilm mass is found to be more susceptible to treatment exposure, while the embedded cell population is more resilient. This susceptibility of the biofilm may also be explained by weaker binding affinity of the cells and the irregularity of the biofilms shape, lacking physical structure, whereas the embedded bacterial cells resilience may be due to Gram negative bacteria’s additional defences.

In comparison, *S. aureus* biofilms were found to have much greater resistance to treatments, even increasing in physical size (most notably to chitosan), but were shown to be quite susceptible to treatments in terms of their metabolic bacterial activity. The external biofilm mass response could support the idea that *S. aureus* biofilm formations are more robust, most likely due to their denser shape and stronger binding affinity. By comparison of the *P. aeruginosa* and *S. aureus* responses to chitosan, it is quite likely that *S. aureus* is incorporating chitosan into its outer matrix, which is certainly aided by its advanced binding ability. This idea is supported by the CV results of the *S. aureus* biofilms reporting significant increases in response following chitosan treatment. As previously reported, chitosan has a high affinity to natural compounds, including dyes such as CV, and is known to hold high binding affinity with bacterial biofilm matrices [63,64]. While it is difficult to ascertain whether the increased response is solely due to the bound chitosan or also involves upregulation of the *S. aureus* biofilm formation in response, it is clear the occurrence is unique to *S. aureus*, as it is not observed to any extent in *P. aeruginosa* (Figure 1C, Figure 2C, Figure 3C, Figure 4C, Figure 5C and Figure 6C).

Another consideration noted from comparison of lid pegs to plate wells as growth surfaces is the physical characteristics of the treatments used. While soluble compounds would not be affected by either growth surface, the use of heavier compounds or insoluble suspensions would raise concerns. For example, exposure during the attachment phase was noted to have most inhibitory effect against *P. aeruginosa* biofilm mass on lid pegs. As this was carried out over a 1-h period, it was most likely still in suspension and able to exhibit this effect. Exposure against *S. aureus* attachment, however, exhibited notable increases in biofilm formation and also bacteria metabolic activity, on both plate wells and lid pegs, but most notably plate wells. There have been studies highlighting ZnO to cause increases in *S. aureus* biofilms at sub-MIC concentrations, which may explain such occurrences [65]. In terms of physical availability, it may be due to *S. aureus* having higher binding affinity, where it will bind ZnO more readily than *P. aeruginosa*, and with ZnO’s physical characteristics. These effects were highlighted in biofilm growth inhibition, with *S. aureus* reporting significant inhibition of metabolic activity on lid pegs. ZnO is known to embed itself into biofilm matrices and formations, where it can then exhibit its inhibitory effects but also aid in cell adhesion through zinc-activated surface protein SasG [66].

### 4.3. Inclusion of Attachment Inhibition to Standardised Anti-Biofilm Assessment

Bacterial attachment is one of the most vital stages of biofilm development, requiring a successful bond between bacterial cells and a suitable surface, leading to EPS secretion, irreversible attachment and subsequent biofilm development [11,12]. As previously mentioned, there have been several studies that have assessed the effects of different antimicrobial compounds and materials against the initial bacterial attachment stage of biofilm development [19,35]. Review of these studies have identified a number of draw backs regarding their methods, such as their lack specificity with treatment exposure.

The methods presented here offer an improved and more thorough means for the study of the anti-biofilm capabilities of test compounds by addressing these issues and building upon known biofilm behaviour and anti-biofilm test methods. As mentioned, there are well-established methods for assessing biofilm inhibition and biofilm eradication (such as the MBIC and MBEC assays), which also make use of the 96-well plate format. The main advantage of such assays is the 96-well format that allows higher through-put analysis of varying compounds/concentrations in a single experiment, but also their similarity to MIC assays allowing those familiar with these techniques to apply them to anti-biofilm studies [45,67,68]. Most steps for the biofilm inhibition and biofilm reduction assays follow those already published; however, additional steps were introduced as a means to standardise certain aspects, such as the use of blood plasma to aid attachment initiation, the use of both the lid peg and plate wells as growth surfaces, and the 1-h attachment period. While attachment has been previously considered in other biofilm studies; it is not consistently used across studies or individually assessed [19,69,70]. Its inclusion is important in such studies as it adds a parameter which further recreates natural biofilm development, wherein many medically associated biofilms are established in dynamic environments with fluid flow, such as on catheters or stents, which do not allow extended time for bacteria to attach [71,72]. Such surfaces are coated by host proteins which then provide the necessary bio-chemical interactions for initial attachment, imitated in this study using blood plasma pre-treatment. Furthermore, this step seeks to remove non-adherent bacteria from the test environment which may collect at the base of plate wells rather than attach and form active biofilms, allowing studies to only include true biofilm bacteria.

## 5. Conclusions

The present study compares the use of lid pegs and microtiter plate wells as attachment and subsequent growth surfaces for bacterial biofilms using *P. aeruginosa* and *S. aureus* as model species. Through the use of crystal violet staining of the biofilm mass and resazurin reduction to indirectly measure internal biofilm embedded bacteria, this informed novel findings between biofilm growth, treatment type, growth surface and bacterial strain. As other published studies have reported, use of both CV and Rez allowed greater data recovery, presenting the value and ease of inclusion of both methods [23,33,73].

The described attachment inhibition stage has been effective in revealing key insights into bacterial behaviour and interactions which would otherwise have been unexplored. The use of treatments to hinder bacterial attachment has produced varied results, further highlighting differences in bacterial species responses, where Gram-negative *P. aeruginosa* exhibited susceptibility to attachment inhibition (particularly to AgNO_3_), whereas Gram-positive *S. aureus* exhibited a much greater resilience (particularly to chitosan). These results identify initial attachment as a promising target for inhibiting *P. aeruginosa* biofilm formations and provide data into the use of both AgNO_3_ and ZnO as effective inhibitors, including concentration ranges to assess, which could development into a synergetic co-treatment of the two compounds, which has been reported previously [74]. The use of both peg lids and plate wells also allowed further insight into bacterial behaviour and biofilm formations. It was noted across all assays that biofilm formations on plate well surfaces were more resilient to treatments, particularly the non-motile *S. aureus*. This gives insight into formation behaviours and final biofilm forms, with lid peg biofilms requiring higher levels or motility but resulting in weaker biofilms, as well as biofilms that allow formation through sheer accumulation rather than active movement and attachment to be more robust and difficult to treat. However, as revealed by CV and Rez comparisons, while the external biofilm may report more resilience or physical mass, which would imply of greater protection, the biofilm-embedded bacteria may remain susceptible to treatment. Some of the reported treatments were more effective at such, which may be credited to their small physical size (nisin, ZnO NPs), allowing permeation into the biofilm structure.

The results of this study have shown AgNO_3_ to be highly effective inhibitor of *P. aeruginosa* bacterial attachment, biofilm growth as well as a successful treatment for the eradication of established biofilms. It was not shown to have major inhibitory effect versus *S. aureus* biofilm development but was noted to exhibit strong effects against bacteria metabolic activity. While nisin was not expected to hold any effect against *P. aeruginosa*, it did report notable inhibition and reduction against biofilm formations. Nisin exhibited moderate effects against *S. aureus*, both its biofilm mass and embedded bacterial populations. Chitosan demonstrated notable inhibition and reduction against *P. aeruginosa* biofilms, but it was found to cause significant increases in *S. aureus* biofilm formations. The opposite was observed against biofilm embedded bacterial populations, with *P. aeruginosa* showing resistance and even higher activity in response, with *S. aureus* populations reporting significant reductions. ZnO was somewhat effective against *P. aeruginosa* biofilm mass, being able to exhibit its effect at attachment and development stages, but unable to affect already formed biofilms. The Rez analysis revealed ZnO to cause an increase in bacteria metabolic activity. Again, in contrast, ZnO was unable to inhibit or reduce *S. aureus* biofilms, instead causing minor increases. However, Rez analysis revealed that it was able to inhibit or reduce biofilm embedded bacterial populations metabolic activity.

The development of a standardised method for biofilm assessment has proven a difficult endeavour, primarily due to the nature of bacterial biofilm development which can vary and alter greatly. The inclusion of an attachment inhibition assessment was a key step in the development of a more informative and standardised approach, as the prevention of bacterial attachment to surfaces should be treated as equally important for anti-biofilm studies. While the use of CV staining and resazurin have been utilised in previous studies, the present study demonstrates the ability to successfully combine both approaches into a single experiment for greater insight underpinning biofilm formation and prevention. Furthermore, while previous resazurin studies used comparisons to plate counts, the present methods allow for a more accurate representation of actual biofilm bacterial counts, while using less materials and resources [8,40]. This study has comprehensively combined and applied numerous biofilm growth, detection and assessment techniques into a single protocol design. The standardised aspects of this combined protocol will inform future biofilm mediated antimicrobial resistance research and innovation, which remains a major issue in healthcare.

## Figures and Tables

**Table 1:** Disruptive Capabilities against Biofilm Mass. Table summarises the effects of bioactives against *P. aeruginosa* and *S. aureus* biofilm mass as determined by crystal violet analysis. Effects are averaged based on their effects against biofilm development at bacterial attachment, biofilm growth inhibition or formed biofilm reduction on both peg lids and plate well walls. Effects are scaled from +++ (High inhibition/reduction) to --- (High promotion/increase), with 0 marking an averaged “no effect”.

	Disruptive Capabilities Against Biofilm Mass					
	Attachment Inhibition		Biofilm Inhibition		Biofilm Reduction	
	*P. aeruginosa*	*S. aureus*	*P. aeruginosa*	*S. aureus*	*P. aeruginosa*	*S. aureus*
AgNO_3_	++++	--	++++	+	+++	+++
Nisin	+	++	0	+	+	++
Chitosan	+	---	+	----	+	----
ZnO	++	--	+	--	0	--

**Table 2:** Disruptive Capabilities against Metabolically Active Biofilm Bacteria. Table summarises the effects of bioactives against *P. aeruginosa* and *S. aureus* biofilm-embedded bacterial populations as determined by resazurin analysis. Exhibited results are averaged based on their effects against final biofilm embedded populations from treatment at bacterial attachment, biofilm growth inhibition or against formed biofilms on both peg lids and plate well walls. Effects are scaled from ++++ (High inhibition/reduction) to ---- (High promotion/increase), with 0 marking an averaged “no effect”.

	Disruptive Capabilities Against Metabolically Active Biofilm Bacteria					
	Attachment Inhibition		Biofilm Inhibition		Biofilm Reduction	
	*P. aeruginosa*	*S. aureus*	*P. aeruginosa*	*S. aureus*	*P. aeruginosa*	*S. aureus*
AgNO_3_	+++	+	+++	+++	++++	++++
Nisin	0	++	0	++	+	+++
Chitosan	+	+	---	0	--	++
ZnO	0	-	---	+	---	+++

## Data Availability

The original contributions presented in this study are included in the article/Supplementary Material. Further inquiries can be directed to the corresponding authors.

## Supplementary Materials

The following supporting information can be downloaded at: https://www.mdpi.com/article/10.3390/microorganisms14061238/s1, Figure S1: Results of crystal violet staining.

## Abbreviations

The following abbreviations are used in this manuscript:

AMR	Antimicrobial Resistance
BMR	Biofilm Mediated Resistance
CV	Crystal Violet
EPS	Extracellular Polymeric Substances
GC	Growth Control
MAIC	Minimum Attachment Inhibitory Concentration
MBEC	Minimum Biofilm Eradication Concentration
MBIC	Minimum Biofilm Inhibitory Concentration
Rez	Resazurin
RT	Room Temperature
SC	Sterility Control
