# Supplementary material for: A Standardised Combinational Method for Evaluating Antimicrobial Compounds Against Biofilm Attachment, Development and Eradication
Source: Microorganisms. 2026 May 30;14(6):1238. doi: 10.3390/microorganisms14061238 (PMC13303750; doi:10.3390/microorganisms14061238)
Supplement: Supplementary file 1 [file microorganisms-14-01238-s001.zip › microorganisms-4202516-supplementary.pdf]

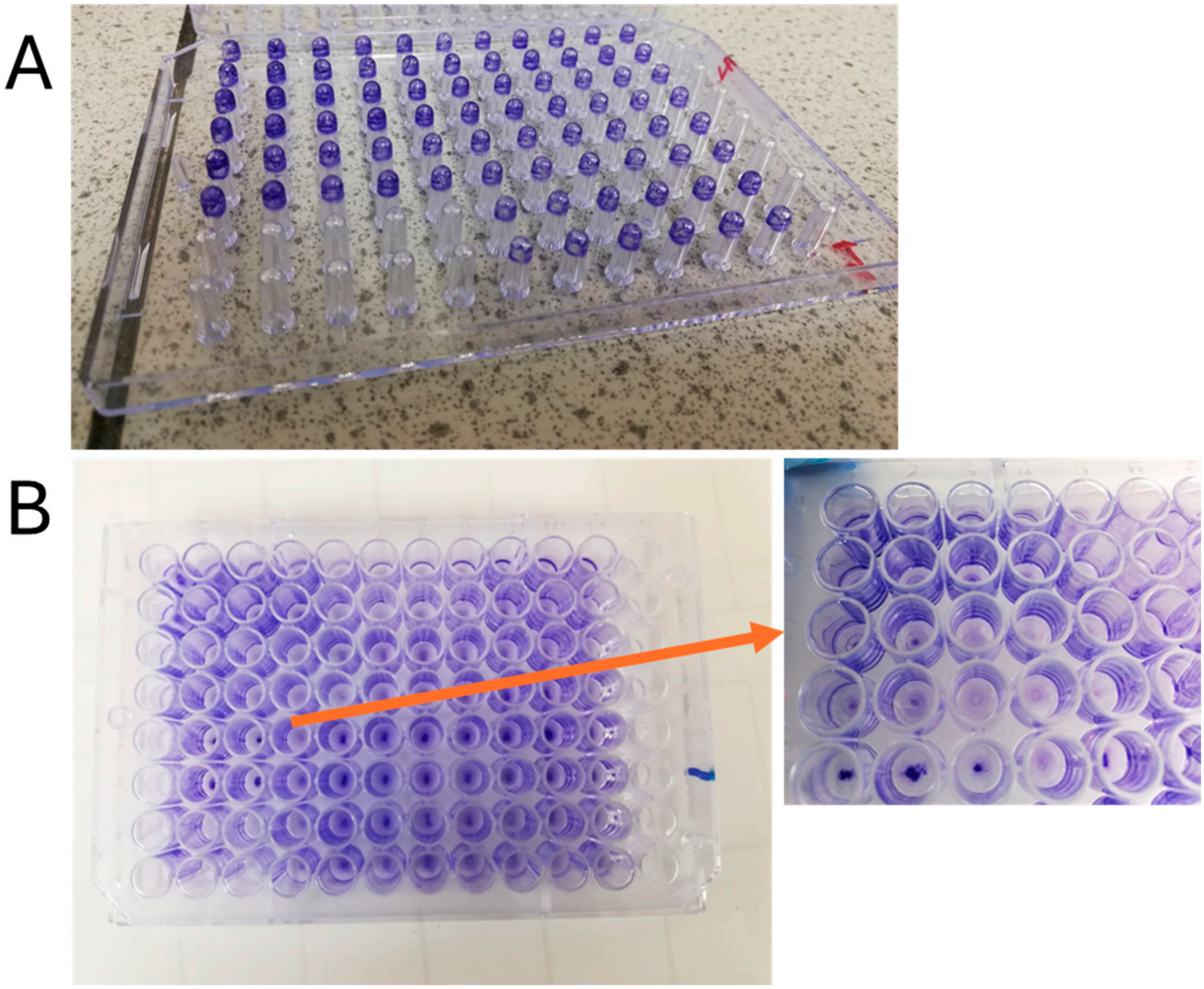

Figure S1: Results of Crystal Violet staining. Figure shows images of biofilm formations on **A**: peg lids and **B**: plate wells following heat fixing and crystal violet (CV) staining or biofilms.
